# *CpxA/R*‐Controlled Nitroreductase Expression as Target for Combinatorial Therapy against Uropathogens by Promoting Reactive Oxygen Species Generation

**DOI:** 10.1002/advs.202300938

**Published:** 2023-07-05

**Authors:** Hao Ren, Zixing Zhong, Shuang Zhou, Yiyang Wei, Yujiao Liang, Huiling He, Zijian Zheng, Mengyuan Li, Qian He, Tengfei Long, Xinlei Lian, Xiaoping Liao, Yahong Liu, Jian Sun

**Affiliations:** ^1^ Guangdong Laboratory for Lingnan Modern Agriculture National Risk Assessment Laboratory for Antimicrobial Resistance of Animal Original Bacteria College of Veterinary Medicine South China Agricultural University Guangzhou 510642 China; ^2^ Guangdong Provincial Key Laboratory of Veterinary Pharmaceutics Development and Safety Evaluation South China Agricultural University Guangzhou 510642 China; ^3^ Jiangsu Co‐Innovation Center for the Prevention and Control of Important Animal Infectious Disease and Zoonoses Yangzhou University Yangzhou 225009 China

**Keywords:** combinatorial therapy, cpx two‐component system, nitroreductase, reactive oxygen species, uropathogens

## Abstract

The antibiotic resistances emerged in uropathogens lead to accumulative treatment failure and recurrent episodes of urinary tract infection (UTI), necessitating more innovative therapeutics to curb UTI before systematic infection. In the current study, the combination of amikacin and nitrofurantoin is found to synergistically eradicate Gram‐negative uropathogens in vitro and in vivo. The mechanistic analysis demonstrates that the amikacin, as an aminoglycoside, induced bacterial envelope stress by introducing mistranslated proteins, thereby constitutively activating the *cpxA*/*R* two‐component system (Cpx signaling). The activation of Cpx signaling stimulates the expression of bacterial major nitroreductases (*nfsA*/*nfsB*) through *soxS*/*marA* regulons. As a result, the *CpxA/R*‐dependent nitroreductases overexpression generates considerable quantity of lethal reactive intermediates via nitroreduction and promotes the prodrug activation of nitrofurantoin. As such, these actions together disrupt the bacterial cellular redox balance with excessively‐produced reactive oxygen species (ROS) as “Domino effect”, accelerating the clearance of uropathogens. Although aminoglycosides are used as proof‐of‐principle to elucidate the mechanism, the synergy between nitrofurantoin is generally applicable to other Cpx stimuli. To summarize, this study highlights the potential of aminoglycoside‐nitrofurantoin combination to replenish the arsenal against recurrent Gram‐negative uropathogens and shed light on the Cpx signaling‐controlled nitroreductase as a potential target to manipulate the antibiotic susceptibility.

## Graphical abstract

The nitrofurantoin is found to synergize with aminoglycosides to eradicate Gram‐negative pathogens. The mechanistic study reveals that aminoglycosides induced protein misfolding to constitutively upregulate nitroreductase expression via Cpx two‐component system. The overexpression of nitroreductases promotes nitrofurantoin prodrug activation and lethal reactive oxygen species accumulation, which collectively eliminate pathogens as a synergistic ‘Domino effect’.

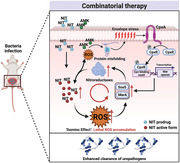

## 1 Introduction

In modern society, the urinary tract infection (UTI) has become one of the most common bacterial infection, affecting the health of ≈150 million people globally.^[^ ^1^ ^]^ With the population aging, socioeconomic development and advances in disease prevention and control, the clinical significance of UTI received special attentions as such diseases negatively impact individuals by impairing their quality of life to a larger extent.^[^ ^2^ ^]^ Both Gram‐negative and Gram‐positive bacteria were found as causative agents for incidence of UTIs, where the uropathogenic *Escherichia coli* (UPEC) is the most prevalent followed by *Klebsiella pneumoniae*, *Enterococcus faecalis*, *Proteus mirabilis*, and some fungi.^[^ ^3^ ^]^ The previous studies have outlined that these uropathogens develop intricate mechanisms to invade, colonize, adapt, persist, and disseminate in host, eventually establish the infections in urinary tracts.^[^ ^4^ ^]^ Most UTIs are initiated when uropathogens move into urinary tract through the urinary meatus before ascending up into the bladder lumen and kidney, where these pathogens cause the clinical signs and symptoms of pyelonephritis.^[^ ^5^ ^]^ In such cases, the uropathogens are able to enter and spread into the bloodstream if without proper and efficient treatments.^[^ ^6^ ^]^ Particularly in the patient with indwelling catheter, the morbidity and mortality of UTI have increased with higher chance of secondary bloodstream infections.^[^ ^7^ ^]^ Of note, the UTI‐induced bacteremia can lead to life‐threatening outcomes when the concurrent systemic inflammation presented.^[^ ^8^ ^]^ Thus, the appropriate therapeutics and prophylactics are necessary and important to curb UTI before the systematic infection.

In view of therapeutics and prophylactics against UTI, the current treatment paradigms reply on usage of broad‐spectrum antibiotics as drug of choice.^[^ ^9^ ^]^ Such antibiotics (e.g., sulfonamides, fluoroquinolones, *β*‐lactam, aminoglycosides, and nitrofurans) have been successfully used to combat UTI but their efficacies are severely challenged by continued emergence of antibiotic resistance, especially the extended‐spectrum *β*‐lactamase (ESBL)–producing Gram‐negative bacteria.^[^ ^10^ ^]^ These multidrug‐resistant (MDR) uropathogens are increasingly found in clinics, consequently leading to accumulative treatment failures and recurrent episodes of UTI.^[^ ^11^ ^]^ And lamentably, the traditional source of antibiotic was seemingly overmined, as the discovery of new antibiotics has been hanging far behind the rapid evolution of antibiotic resistances.^[^ ^12^ ^]^ Therefore, the alternative approaches are of urgent needs to tackle the MDR uropathogens and prevent the recurrences of UTI.^[^ ^13^ ^]^ To cope with this, the alternatives including combination strategy, drug repurposing, anti‐virulence therapy, rational optimization of leads or new leads development from untapped source, have been introduced and significantly expanded our arsenal to combat the bacterial infection.^[^ ^14^ ^]^ For instance, Spaulding and colleagues developed an antiviral mannoside that targets the pilus of pathogenic *E. coli* to curtail the colonization of genetically diverse UPEC isolates and prevent the recurrent UTI.^[^ ^15^ ^]^ Among the few newly‐introduced antibiotics, the chimeric antibiotics synthesized by Luther et al. was found to exert bactericidal effect to pathogens including *P. aeruginosa* and *Enterobacteriaceae* by binding to both lipopolysaccharide (LPS) and the *β*‐barrel folding complex.^[^ ^16^ ^]^ However, all these approaches have pros and cons, the cost, biosafety, efficacy, specificity as well as the side effects should be carefully considered. Comparing with development of new antibiotics, the combination strategy provides a promising approach to revitalize the “old drugs” with balance in efficiency, cost and time‐consumption.^[^ ^17^ ^]^ Such combination therapy, exemplified by the combination of *β*‐lactam antibiotic with *β*‐lactamase inhibitors like clavulanate, has shown great potential to be effective against a broad range of uropathogens.^[^ ^18^ ^]^ However, many of established drug combinations, especially the early attempts, were assembled on an ad hoc basis with limited rigorous understanding of molecular mechanisms.^[^ ^19^ ^]^ Without in‐depth understanding of mechanistic information, most combination of antibiotics are given in an empirical manner.^[^ ^20^ ^]^

In the current study, we found that the combination of amikacin (AMK) and nitrofurantoin (NIT) synergistically eradicated the Gram‐negative uropathogens in vitro and in vivo. Further mechanistic analysis demonstrated that the ribosome‐targeting AMK constitutively activates the two‐component system *cpxA*/*R* via inducing protein misfolding, by which leads to the overexpression of bacterial major nitroreductases through *soxS*/*marA* regulon. The increased nitroreductases promoted the prodrug activation of NIT and accumulation of lethal reactive oxygen species (ROS) to inevitably kill the bacteria. The present study provides AMK‐NIT combination as a feasible therapeutic regimen against UTI and shed the light on the strategy to potentiate NIT through manipulating Cpx signaling.

## 2 Results

### 2.1 AMK Synergizes with NIT to Eradicate Gram‐Negative Pathogens In Vitro and In Vivo

**Figure 1:**
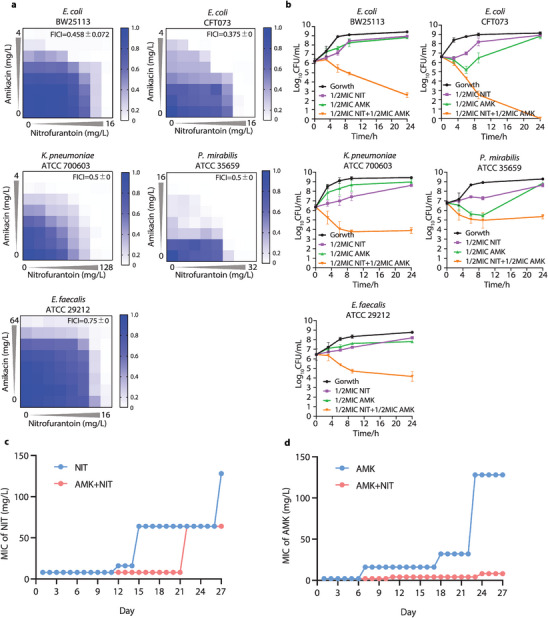
AMK synergized with NIT to eradicate uropathogens in vitro. a) Synergistic interaction between AMK and NIT on representative uropathogens (synergistic interaction was defined as fractional inhibitory concentration index (FICI) of ≤ 0.5); b) Time‐dependent killing of uropathogens by the combination of AMK and NIT (data were presented as mean ± SD, *n* = 3); c) Combinatorial therapy of AMK and NIT prevented the evolution of resistance to NIT in vitro. d) Combinatorial therapy of AMK and NIT prevented the evolution of resistance to AMK in vitro.

**Figure 2:**
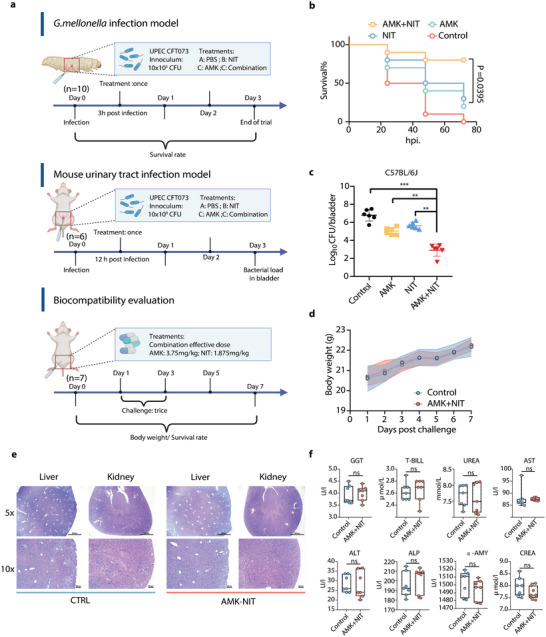
The in vivo efficacy and biocompatibility of AMK‐NIT combination. a) Scheme of the experimental protocol of animal trials; b) Survival curve of infected larva with different treatments (*n* = 10); c) Bacterial load in bladders of infected mice with different treatments (*n* = 6); d) Body weight of healthy mice (*n* = 7 per group) received PBS (control, 100 µL) and AMK‐NIT combination (AMK: 3.75 mg kg^−1^, 50 µL, NIT: 1.875 mg kg^−1^, 50 µL); e) H&E staining of livers and kidneys from healthy mice (*n* = 7 per group) received (control, 100 µL) and AMK‐NIT combination (AMK: 3.75 mg kg^−1^, 50 µL, NIT: 1.875 mg kg^−1^, 50 µL); f) Serum indices of healthy mice (*n* = 7) received PBS and AMK‐NIT combination, GGT: Gamma‐glutamyltransferase, T‐BILL: Total Bilirubin, UREA: Urea, AST: Aspartate Transferase, ALT: Alanine Transaminase, ALP: Alkaline Phosphatase, *α*‐AMY: *α*‐Amylase, CREA: Creatinine. All data are presented as mean ± SD and the significances were determined by one‐way ANOVA (* *p* < 0.05, ** *p* < 0.01, *** *p* < 0.001, ns: not significant)

In our previous work, we identified the combination of AMK with NIT as an effective polytherapy against some clinical *E. coli* isolates.^[^ ^21^ ^]^ As both AMK and NIT are known to be used for treatment of urinary tract infection (UTI) because of their rapid and high urinary excretion,^[^ ^22^ ^]^ here we extended our scope to test whether AMK‐NIT combination is generally applicable to combat against the urinary pathogens. Accordingly, a range of common urinary pathogens including *E. coli*, *K. pneumoniae*, *P. mirabilis* and *E. faecalis* were selected as representatives to determine the efficacy of the combination. The checkerboard assay showed strong synergistic interaction was observed between AMK and NIT to all selected bacteria except *E. faecalis* (**Figure** **1**a). In addition to the checkerboard assay, an assessment for bactericidal activity would further our in‐depth understanding toward the synergism between AMK and NIT. Analogously, both AMK and NIT demonstrated relatively weak bactericidal activity at 1/2 of their minimum inhibitory concentration (MIC). Whereas, the combination of AMK and NIT at the same concentration displayed significantly improved bactericidal activity to selected bacteria, among which the *E. coli* was seemingly more susceptible to the killing by the AMK‐NIT combination (Figure 1b). Since the potential to curb the antibiotic resistance evolution is clinically significant, we further detected the resistance evolution in bacteria treated by AMK, NIT or their combination. The development of resistance to AMK or NIT was observed to be reduced by the application of combination, whereas the AMK or NIT alone rapidly resulted in a striking increment of MIC especially for NIT (Figure 1c,d). In view of the promising synergism of AMK‐NIT on the Gram‐negative uropathogens in vitro, we next tested its in vivo efficacy and biocompatibility as schematically illustrated in **Figure** **2**a. First, *G. mellonella* killing model was employed to determine the protection against the lethality of pathogens in infected larva. The combination of AMK and NIT showed strikingly increased survival rate comparing with their usage as alone (Figure 2b). Then, a mice urinary infection model was established to evaluate the clearance of uropathogens. Cheerfully, the polytherapy of AMK and NIT eradicated the infected pathogen in a highly efficient manner, 2‐log_10_ extra colony forming units (CFU) bacterial load was reduced comparing to the monotherapy based on AMK or NIT alone (Figure 2c). Eventually, we analyzed the biocompatibility of AMK‐NIT combination to elucidate their potential side effects. However, neither mortality nor significant differences were observed in the body weight of the treated mice when compared with the untreated controls (Figure 2d; Figure S1, Supporting Information). The hematoxylin and eosin (H&E) staining revealed no obvious abnormalities in livers and kidneys of the mice received combinatorial therapy (Figure 2e). In addition, the serum indices were maintained at normal physiological levels, indicating that the combinatorial therapy did not induce hepatotoxicity or nephrotoxicity in vivo (Figure 2f). These encouraging results suggested that the combination of AMK and NIT synergistically tackled the uropathogens in vitro and in vivo and the underlying mechanism is of great interest to be further deciphered.

### 2.2 Accumulation of Bactericidal ROS Defines the Synergy of Combinatorial Therapy

**Figure 3:**
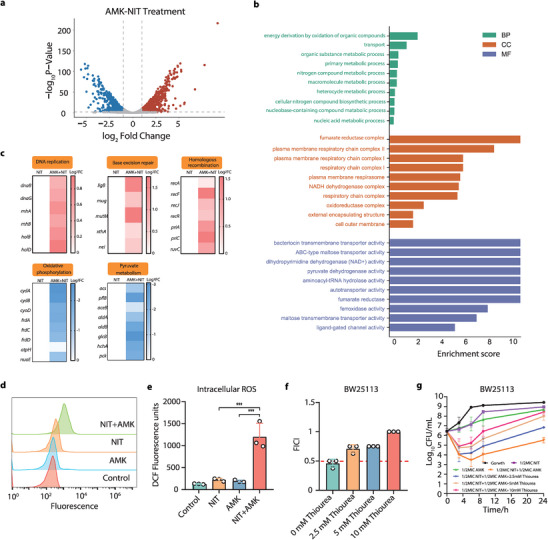
Transcriptomic analysis reveals that accumulation of bactericidal ROS accounted for the synergy between NIT and AMK. a) Volcano plot of the differential expression genes (DEGs) in bacteria treated by combination of AMK and NIT; b) Gene ontology annotation analysis of the DEGs in bacteria treated by combination of AMK and NIT; c) Selected DEGs involved in DNA replication, Base excision repair, Homologous recombination, Oxidative phosphorylation and Pyruvate metabolism; d) AMK‐NIT combination promoted ROS accumulation revealed by flow cytometry (*n* = 3); e) Quantitative analysis of bacterial intracellular ROS; f) ROS scavenger thiourea (2.5 to 10 mm) abolished the synergy of AMK and NIT in a dose‐dependent manner (*n* = 3); g) Time‐dependent killing of bacteria by the combination of AMK and NIT in presence or absence of thiourea (*n* = 3, 2.5 to 10 mm). All data are presented as mean ± SD and the significances were determined by T‐test (* *p* < 0.05, ** *p* < 0.01, *** *p* < 0.001).

To explore the underlying mechanism for the observed synergy, the transcriptomic profiles of bacteria were investigated after exposure to single application of NIT, AMK as well as their combinations for 4 h. The application of AMK or NIT alone affected the ribosome functions and several metabolic pathways (Figure S2, Supporting Information). Of note, the combination globally reprogramed the transcriptome of the bacteria in comparison with using NIT alone by inducing the up‐regulation of 732 genes and downregulation of 499 genes respectively (**Figure** **3**a). Furthermore, the enrichments analysis that the differential expression genes (DEGs) were enriched in pathways involved in macromolecule metabolic process, respiratory chain complex, cell outer membrane and etc. Intriguingly, we found that in the combination groups, the pathways involved in repair functions like base excision repair, homologous recombination and DNA replications were significantly enriched at the expense of reduced bacterial metabolism (pyruvate metabolism) and energy supply (oxidative phosphorylation) (Figure 3b,c). It has been well established that the prokaryotes generally suppress their metabolic activities and activate the repair systems as mechanisms of defense in response to the oxidative stress.^[^ ^23^ ^]^ Therefore, we hypothesized that these pathways were regulated to offset the excessive oxidative damage, which plausibly caused by the combination of AMK with NIT and explains the synergy therein. To validate our hypothesis, we next assessed the intracellular ROS accumulation of bacteria after different treatments using a ROS‐sensitive dye. The results demonstrated that, comparing with using AMK or NIT alone, the combination of both resulted in considerable quantity of ROS generated within the bacterial cells (Figure 3d), as ∼6‐fold increment of fluorescence was observed (Figure 3e). Except the model strain used in the mechanistic study, the AMK‐NIT combination also substantially promoted ROS generation in CFT073, the pathogen used in animal trials (Figure S3, Supporting Information). Since the accumulative ROS was posited to play an essential role in the synergistic interaction between AMK and NIT, we therefore probed the synergistic inhibition of the AMK‐NIT combination when the ROS was scavenged by thiourea. As shown in Figure 3f, the exogenous addition of thiourea abolished the synergy of the combination in a concentration‐dependent manner (details in Figure S4, Supporting Information). The same phenomenon was also observed in the time‐killing assay (Figure 3g), the bactericidal activity of the combination was profoundly weakened in tandem with elevated addition of thiourea from 2.5 to 10 mm. Collectively, these data suggested that the combined usage of AMK and NIT was able to substantially promote the ROS production, which is responsible for the synergy to eradicate the bacteria in a highly efficient fashion.

### 2.3 Overexpression of Nitroreductases is Central to Synergistic Interaction

**Figure 4:**
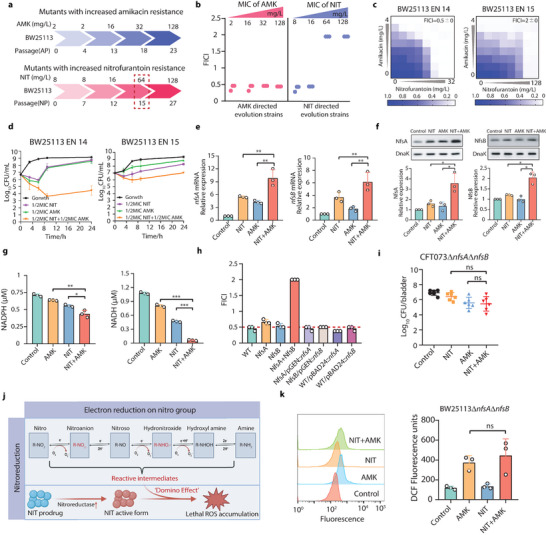
Directed evolution reveals that combinatorial therapy‐mediated nitroreductase overexpression defines synergism by perturbing cellular redox balance. a) Directed evolution of bacteria in presence of AMK or NIT; b) NIT resistance diminished the synergy of AMK‐NIT combination; c) Mutation in bacterial nitroreductases led to abatement of synergy between AMK and NIT (*n* = 3); d) Reduced bactericidal activity of AMK‐NIT combination was observed on strains with mutation in bacterial nitroreductases (*n* = 3); e) AMK‐NIT combination promoted expression of bacterial major nitroreductases at transcriptional level; f) AMK‐NIT combination promoted expression of bacterial major nitroreductases at translation level (*n* = 3); g) Over‐activated cellular nitroreduction led to depletion of NADH and NADPH in bacteria treated by AMK‐NIT combination (*n* = 3); h) Loss of function in nitroreductases abolished the synergism between AMK and NIT in vitro (*n* = 3); i) Loss of function in nitroreductases abolished the synergism between AMK and NIT in vivo (*n* = 6); j) *CpxA/R*‐dependent nitroreductase overexpression promoted “Domino effect” in ROS accumulation via nitrofurantoin activation and nitroreduction; k) Loss of function in nitroreductases failed to trigger the ROS overload (*n* = 3). All data are presented as mean ± SD and the significances were determined by T‐test (in vitro assays) or one‐way ANOVA (in vivo assays, where the * *p* < 0.05, ** *p* < 0.01, *** *p* < 0.001).

With confirmed role of ROS in synergistic interaction between AMK and NIT, the logical next step is to explore the underlying mode of action to trigger the excessive ROS production. To this end, the bacterial directed evolution was performed to observe whether evolved resistance to single drug alters the response of bacteria to polytherapy. After serial passage in medium supplemented with antibiotics, the bacteria evolved to be highly resistant to AMK and NIT, with MIC increased by 64‐ and 16‐fold respectively. It was interesting to find that, the AMK‐NIT combination remained their synergy to AMK‐resistant subpopulations, even the MIC of them increased to 128 µg mL^−1^. In the contrast, the evolved NIT resistance abrogated the synergism of AMK‐NIT combination as the FICI increased to 2 (**Figure** **4**a,b; details in Figure S5, Supporting Information). Since the FICI rapidly arose in the *E. coli* in NIT‐directed passages of 15^th^ generation (EN15, NIT resistant) instead of in 14^th^ generation (EN14, NIT‐susceptible), we speculated that the acquisition of resistance to NIT readily abolished the synergy. To test this, the subpopulations without (EN14) or with (EN15) NIT resistance in NIT‐directed passage were subjected to the checkerboard assay and time‐killing assay. The results demonstrated that the acquisition of NIT resistance dampened the synergistic interaction, by which led to increased FICI and bacterial survivors in killing assay (Figure 4c,d). These data conceivably suggested that the NIT played major role in the synergy and the combination to AMK aided NIT to improve its efficacy than being used alone. We then performed the whole genome sequencing on these passage strains and found the mutations in *nfsA* and *nfsB* (Figure S6, Supporting Information), the major genes encoding the nitroreductase. The cytosolic nitroreductases are the enzymes known to activate the prodrug of nitrofurantoin to its bactericidal form via nitroreduction in bacterial cell,^[^ ^24^ ^]^ and it makes sense that the AMK potentiate NIT efficacy through regulating nitroreductase. Thus, we probed the expression of major nitroreductases, namely *nfsA* and *nfsB* in bacteria treated by AMK, NIT, and their combination. Of no surprise, the combination of AMK and NIT significantly increased the transcription of both nitroreductases in the bacteria comparing with using AMK or NIT alone (Figure 4e). Consistently, the result of immuno‐blotting also indicated the nitroreductases were cumulated at protein level under treatment of AMK‐NIT combination (Figure 4f). To reinforce the notion that modulation by AMK‐NIT combination on nitroreductase expression is also validated in vivo, a luminescent reporter (pUC‐luxCDABE‐*nfsA*) was constructed by fusing the *lux* gene to the promoter of *nfsA* and used for observing nitroreductase expression in the animal model. As indicated by the detection using in vivo imaging system (IVIS), the over‐expression of nitroreductases was also triggered by the AMK‐NIT combination at in vivo conditions (Figure S7, Supporting Information). These results jointly suggested an overexpression of nitroreductases primed by the AMK‐NIT combination. As the bacterial nitroreductases consume NADH and NADPH as cofactors to catalyze nitroreduction,^[^ ^25^ ^]^ these reducing agents were probed in the bacteria treated by monotherapy and polytherapy. The results supported that the combination of AMK and NIT significantly reduced the level of both NADH and NADPH, suggesting cellular nitroreduction increased in concert with overexpression of nitroreductases (Figure 4g). To decipher the exact role of nitroreductases in synergism of AMK and NIT, the checkerboard assay was conducted on nitroreductase‐deficient strains and their parental strain. The data demonstrated that the deficiency in nitroreductase completely abolished the synergistic interaction between AMK and NIT, which could be compensated by plasmid‐expressed *nfsA* or *nfsB* (Figure 4h; Figure S8, Supporting Information). To explicit whether the over‐expression of bacterial nitroreductase is essential for in vivo synergy of the combination, the monotherapy of AMK or NIT as well as their combination have been applied to treat the mice infected with nitroreductase‐deficient mutant of CFT073. The result exhibited that the combination of AMK and NIT were not able to synergistically eradicate the nitroreductase‐deficient pathogen in vivo, suggesting the critical role of nitroreductase in modulating the synergism between AMK and NIT (Figure 4i). Given that a panel of highly reactive radicals were produced in the successive reactions catalyzed by nitroreductases, it was plausible that the overexpression of *nfsA* and *nfsB* resulted in large quantity of ROS in the bacterial cell through over‐heated nitroreduction. Together with the NIT‐mediated oxidative damage, a synergistic “Domino effect” of ROS production was expected to perturb the bacterial redox balance and enhance the bacterial clearance (Figure 4j). Thereafter, we examined the ROS production in the nitroreductase‐deficient mutant (Δ*nfsA*Δ*nfsB* double mutant) in response to AMK‐NIT combination and found that the AMK‐NIT combination failed to mediate ROS overload in the absence of bacterial nitroreductases (Figure 4k; Figure S9, Supporting Information). Taken together, our data supported that the over‐expression of nitroreductases modulated by the AMK‐NIT combination played the central role in observed synergism, by which prominently generate ROS via nitroreduction and converting NIT to its active form.

### 2.4 AMK‐NIT Combination Promotes the Expression of Nitroreductase via Constitutive Activation of *soxS* and *marA*

**Figure 5:**
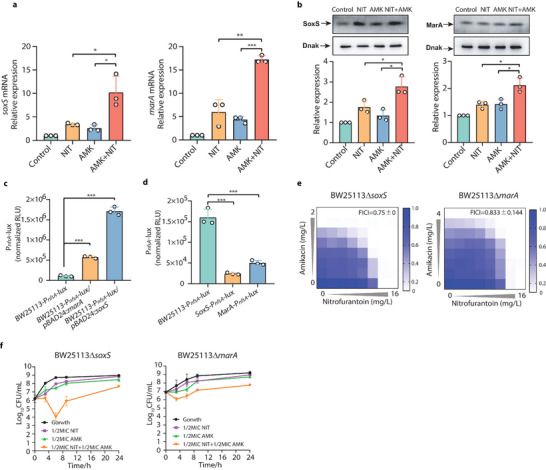
AMK‐NIT combination promotes the expression of nitroreductase via constitutive activation of *soxS* and *marA*. a) AMK‐NIT combination promoted *soxS* and *marA* expression at transcription level (*n* = 3); b) AMK‐NIT combination promoted *soxS* and *marA* expression at translation level (*n* = 3); c) The expression of *nfsA* was under control of *soxS* and *marA* (*n* = 3); d) The AMK‐NIT combination failed to trigger *nsfA* overexpression in the *soxS*‐/*marA*‐deficient mutants (*n* = 3); e) Knockout of *soxS*/*marA* in bacteria abolished the synergy between AMK and NIT (FICI of 0.5–2 was defined as additive or indifference effect); f) Knockout of *soxS*/*marA* in bacteria dampened the bactericidal activity of AMK‐NIT combination (*n* = 3). All data are presented as mean ± SD and the significances were determined by T‐test (* *p* < 0.05, ** *p* < 0.01, *** *p* < 0.001).

Although the information regarding regulation of nitroreductase in bacteria is scarce in previous study, *soxS*/*marA* are the regulons that speculated to control the expression of nitroreductases especially in defined stress conditions.^[^ ^26^ ^]^ Given that both *soxS* and *marA* were found to be significantly up‐regulated in our transcriptomic data, the expression of these two regulators were again quantified by RT‐qPCR. The results were in line with our transcriptomic data, the combination of AMK and NIT eminently increased the transcription of these two genes in comparison with using AMK or NIT alone (**Figure** **5**a). Since both SoxS and MarA protein are sensitive to degradation by Lon protease in the in vitro conditions,^[^ ^27^ ^]^ the effects of AMK, NIT and their combination on translation of these proteins were performed on Lon‐deficient strains (Δ*lon* mutant). Consistently, the results showed that the treatment of AMK‐NIT combination led to augmented translation of both SoxS and MarA protein (Figure 5b). We therefore proposed that *soxS* and *marA* possibly participated in regulating over‐expression of nitroreductases. To confirm this, the luminescent reporter (pUC‐luxCDABE‐*nfsA*) was used to observe how nitroreductase responded to activation of *soxS* and *marA*. As illustrated in Figure 5c, there was a strong luminescent signal induced by activation of *soxS* and *marA*, which supported that the expression of nitroreductase was under control of *soxS*/*marA* regulon. Furthermore, we used the same reporter to estimate the response of nitroreductase to the treatment of AMK‐NIT combination in *soxS* and *marA*‐deficient bacterial strains (Figure 5d). Throughout the experiment, the luminescence was much lower detected in the strains with *soxS* and *marA* defects after co‐incubation with AMK‐NIT combination, suggesting that the combination induced the over‐expression of nitroreductase via activation of *soxS* and *marA*. Subsequently, we performed checkerboard and time‐killing assay on *soxS* and *marA*‐deficient strains and found that the AMK‐NIT combination no longer exhibited synergism in the absence of *soxS* and *marA* regulons (Figure 5e,f). Altogether, these data demonstrated the nitroreductases over‐expression induced AMK‐NIT combination was dependent on constitutive activation of *soxS* and *marA*.

### 2.5 Two‐Component Systems (TCSs) *cpxA/R* Robustly Stimulates *soxS*/*marA* Transcription in Response to Aminoglycoside‐Induced Protein Misfolding

**Figure 6:**
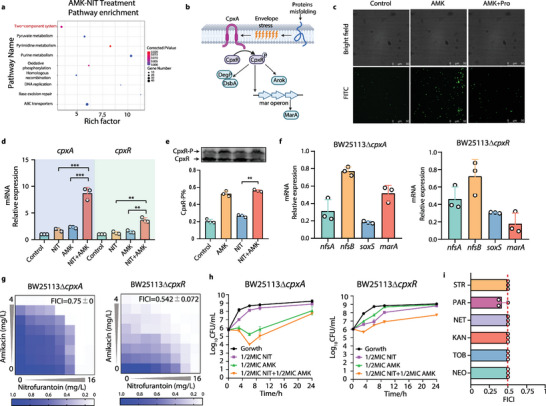
Cpx signaling robustly stimulates *soxS*/*marA* transcription in response to AMK‐induced protein misfolding. a) Pathways involved in two‐component systems (TCSs) were enriched in the bacteria exposed to AMK‐NIT combination or AMK alone; b) TCS *cpxA*/*R* was activated to regulate gene expression in response to envelope stress; c) AMK induced protein misfolding to bacterial cells; d) AMK‐NIT combination promoted the expression of *cpxA* and *cpxR* (*n* = 3); e) AMK‐NIT combination and AMK alone stimulated the phosphorylation of CpxR (*n* = 3); f) Knockout of *cpxA* and *cpxR* blocked the responses of *nfsA*, *nfsB*, *soxS* and *marA* to AMK‐NIT combination (*n* = 3); g) The synergy of AMK and NIT was abolished in the mutants lack of *cpxA* and *cpxR* (FICI of 0.5–2 was defined as additive or indifference effect); h) Synergistically bactericidal activity of AMK‐NIT combination was diminished in the mutants lack of *cpxA* and *cpxR* (*n* = 3); i) Synergistic interaction between NIT with other aminoglycosides (*n* = 3), STR: Streptomycin, PAR: Paromomycin, NET: Netilmicin, KAN: Kanamycin, TOB: Tobramycin, NEO: Neomycins. All data are presented as mean ± SD and the significances were determined by T‐test (* *p* < 0.05, ** *p* < 0.01, *** *p* < 0.001).

Considering that the over‐expression of nitroreductases was due to activation of *soxS* and *marA*, we next sought to investigate how AMK‐NIT combination fine‐tunes the activation of *soxS* and *marA*. Interestingly, from our transcriptomic data, we observed a significant enrichment of the pathways involving TCSs when bacteria exposed to AMK alone or AMK‐NIT combination (**Figure** **6**a). The TCSs are the systems of multi‐step signal transduction pathways encompassing a sensor histidine kinase that detects a specific signal and a cognate response regulator that modulates the signal response once phosphorylated by the sensor protein.^[^ ^28^ ^]^ By analyzing the transcriptomic profile, we found that the genes under regulation of TCS *cpxA*/*R* were drastically reprogrammed (Figure 6b; Figure S10, Supporting Information). The TCS *cpxA*/*R* generally respond to stresses that affect protein folding within the envelope of bacteria.^[^ ^29^ ^]^ It is reasonable that, as a member of aminoglycoside antibiotic, AMK possibly drives the activation of *cpxA*/*R* by inducing protein misfolding to bacterial envelope. Hence, we first employed fluorescein isothiocyanate (FITC) to target the protein aggregates for testing the protein misfolding by AMK in bacterial cell.^[^ ^30^ ^]^ The microscopy analysis revealed that application of AMK promoted the formation of protein aggregates (Figure 6c), which were disaggregated by the chemical chaperone proline .^[^ ^31^ ^]^ Consistent with this observation, alleviating protein misfolding using proline also abolished the synergistic action between AMK and NIT (Figure S11, Supporting Information), implying protein misfolding caused by AMK acts as the physiological cue that affects the downstream regulation. Then, we next probed the transcription of sensor *cpxA* and its cognate regulator *cpxR*. The results showed that the usage of AMK‐NIT combination significantly promoted the expression level of both *cpxA* and *cpxR* (Figure 6d). It was in agreement with prior study that the phosphorylated CpxR protein binds to the DNA of *cpxA* and *cpxR*, thereby to regulate their transcription perse.^[^ ^32^ ^]^ As mentioned above, the functionality of CpxR replies on phosphorylation of regulatory subdomain.^[^ ^33^ ^]^ In this regard, the phosphorylation of CpxR was analyzed on the Phos‐tag gel, where the phosphorylated CpxR and its nonphosphorylated counterpart were able to be separated by using gel‐based electrophoresis.^[^ ^34^ ^]^ The phosphorylated CpxR will show a retarded migration at positions of higher molecular weight on the gel compared with its nonphosphorylated counterpart. The results demonstrated the phosphorylated form of CpxR reached 60% in the bacteria treated with the AMK‐NIT combination yet only 25% of it was phosphorylated by treatment of NIT alone (Figure 6e). Of note, lack of *cpxA* and *cpxR* retarded the transcription of *nfsA*, *nfsB*, *soxS*, and *marA* stimulated by the combinatorial therapy based on AMK‐NIT combination, suggesting *cpxA*/*R* acted as a mediator for over‐expression of such genes in response to AMK‐associated envelope stress (Figure 6f). To see whether activated Cpx signaling directly modulated the transcription of *marA*/*soxS* expression, the electrophoretic mobility shift assay (EMSA) was carried out. Since the promoters of the *mar*/*sox* regulon of *E. coli* contain a binding site (*marbox*) for the homologous transcriptional regulation,^[^ ^35^ ^]^ we examined the binding interaction of purified CpxR to *marbox* motif. As shown in Figure S12 (Supporting Information), the purified CpxR binds to *marbox*, thereby confirming the CpxR exerted direct modulation on *marA*/*soxS* expression via binding their promoters. To ultimately confirm the role of Cpx signaling in the synergy, we tested the synergism of AMK‐NIT combination on these *cpxA*/*R*‐deficient strains. As indicated by the checkerboard and time‐killing assays, loss‐of‐function in Cpx signaling diminished the synergistic interaction between AMK and NIT (Figure 6g,h). Since the activation of Cpx signaling was attributed to AMK‐induced envelope stress, we finally assayed the combination of NIT with a panel of aminoglycosides of clinical use, to investigate whether this synergistic mechanism is generally applicable to NIT with other aminoglycoside agents. As expected, the NIT exhibited extraordinary synergy with a broad spectrum of aminoglycosides including streptomycin (STR), paromomycin (PAR), netilmicin (NET), kanamycin (KAN), tobramycin (TOB), and neomycin (NEO) as shown in Figure 6i. These finding collectively depicted that AMK, as well as other aminoglycosides, potentiated the NIT efficacy by inducing the protein misfolding to bacterial cell, which consequently promoted nitroreductases overexpression via *cpxA*/*R*‐*soxS*/*marA* regulatory cascade.

## 3 Discussion

According to a latest epidemiological study, the global incidence of UTI has prominently increased in recent decades.^[^ ^2^ ^]^ As introduced above, the rising antibiotic resistance, in concert with our understanding toward adverse effects of excessive usage of antibiotic, has underscored the weakness and limitation on our current UTI treatment paradigms.^[^ ^36^ ^]^ Particularly with the bottleneck in introducing novel antibiotics, reinvigorating our existing antibiotics offers a superior opportunity to extend the life of well‐researched and clinically validated drugs for filling the void of antibiotic discovery.^[^ ^37^ ^]^ In this study, we found that the combination of AMK and NIT synergistically eliminated the uropathogens in vitro and in vivo. Fortunately, both AMK and NIT have long history to treat UTI, although their efficacy as monotherapy are challenged by surging prevalence of corresponding resistances.^[^ ^38^ ^]^ This means both drugs enjoy their generally‐recognized safety and approval status for treatment against UTI as defined previously, suggesting their combination is highly promising and feasible for clinic application. Another critical benefit for combination therapy underlined less possibility to establish resistance.^[^ ^12^ ^]^ The combination therapies are able preserve clinical outcomes while reducing unnecessary antimicrobials exposure.^[^ ^39^ ^]^ As elucidated by our data, the combination of AMK and NIT at half dose already accelerated the bacterial clearance from host with high bacterial load. Given that the resistance is expected to be rapidly developed to the therapy only hit one target, the combinations make more targets available, thereby the bacteria are not easy to develop resistance.^[^ ^40^ ^]^ This is consistent with our finding since the de novo resistance was soon observed in the bacteria treated with AMK or NIT alone, yet took place rather slowly under treatment of their combination (Figure 1c). Intriguing, we also found that the combination remained synergism even in the subpopulation established the AMK resistance, demonstrating the plasticity in treating the resistant clinical isolates. These results demonstrated that the combination of AMK and NIT are active against the uropathogens in a synergistic manner and of great feasibility to eradicate UTI in clinic.

**Figure 7:**
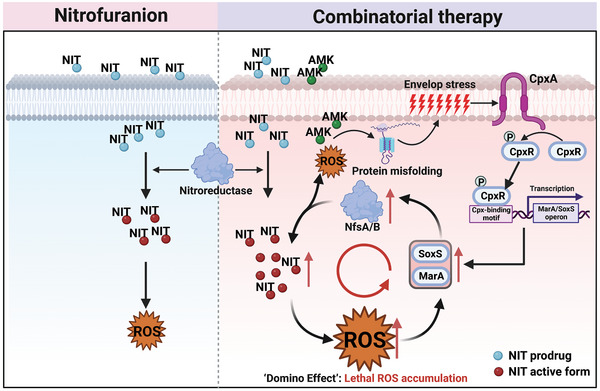
Mechanistic insight into synergistic interaction between AMK and NIT.

Although the combinatorial usages of antibiotics have been extensively reported, only a handful of them clearly elucidated the underlying mechanisms. A well‐established mechanism spotlighted on the strategy using antibiotics with membrane‐acting compounds to increase their uptake, whereby overcoming the intrinsic resistance.^[^ ^41^ ^]^ For instance, colistin was found to potentiate the efficacy of antibiotics targeting protein and RNA synthesis, by promoting intracellular accumulations of such drugs via permeabilizing the membrane.^[^ ^42^ ^]^ In our case, we described a unique synergistic mechanism instead of targeting bacterial membrane (**Figure** **7**). The bactericidal activity of NIT involved in damage to DNA or ribosomes, but exact mode of action remains poorly understood.^[^ ^43^ ^]^ However, the intracellular enzymatic activation from the prodrug to active form is a prerequisite for NIT efficacy, which depends on processing by bacterial nitroreductases.^[^ ^44^ ^]^ As a result, the expression of bacterial major nitroreductases (*nfsA* and *nfsB*) defines the NIT susceptibility. The mutational deactivation of *nfsA* or *nfsB* alone has limited impact on NIT susceptibility, yet the double‐mutant of both confers high‐level resistance to NIT.^[^ ^45^ ^]^ This is similar to our observation on AMK‐NIT synergy, which was completely abolished in the *nfsA*‐*nfsB* double mutants, but to a lesser extend in the single‐gene deletion mutants. Despite in activating NIT into its active form, the nitroreduction catalyzed by nitroreductases is able to generate highly reactive byproducts (Figure 4j) including the ROS produced by oxidation of formed amines.^[^ ^46^ ^]^ Considering the nitroreductases consume NADH and NADPH as cofactor for catalysis, these cellular reducing agents were depleted to a larger extent together with such lethal intermediates. Such perturbation on bacterial redox balance by nitroreduction are able to synergize with NIT‐associated oxidative damage, a “Domino effect” of ROS production to kill the uropathogens is conceivably expected. We can, therefore, conclude that the overexpression of *nfsA*/*nfsB* for NIT prodrug activation and ROS accumulation account for the synergism of the combinatorial therapy. There have been a plenty of publications addressed the active role of ROS formation in mediating bacterial cell death in addition to the primary drug–target interactions.^[^ ^47^ ^]^ The reactive metabolic byproducts, including but not limited to ROS are reportedly to contribute to lethality of antimicrobials by rapidly and largely damaging the essential cellular components via mechanistically different mechanisms.^[^ ^48^ ^]^ Besides damaging the bacterial cell directly, ROS is able to destruct the iron–sulphur clusters in proteins, thereafter releasing the Fenton‐ready iron in ferrous form.^[^ ^49^ ^]^ The accumulation of intracellular ferrous iron and ROS are known as the hallmarks of ferroptosis.^[^ ^50^ ^]^ Although the concept of ferroptosis is more well‐explicated in mammalian cells, recent studies highlighted the occurrence of ferroptotic‐like damages in bacterial cells in response to defined conditions.^[^ ^51^ ^]^ Hence, the ferroptosis‐like death under overwhelming production of ROS is also prone to be presented by the combinatorial therapy, by which exerts deleterious effect to pathogens for enhanced eradication. And the multifaceted impacts of excessive ROS deserve special attention in the future investigation to better design and optimize the antimicrobial agents. Our latter experiments evidenced that AMK promoted the *nfsA*/*nfsB* expression through regulating Cpx signaling. The TCS *cpxA*/*R* sense the envelope stress and regulate the response, yet is only common among the gamma proteobacteria.^[^ ^52^ ^]^ This explained why the AMK‐NIT combination was only found to be synergistic in uropathogens belonging to *Enterobacteriaceae*, but not the E*. faecalis* (Figure 1a) whose *cpxA*/*R* is absent. As exemplified in the prior studies, the constitutive activation Cpx singaling have been associated with intrinsic antibiotic resistances to *β*‐lactams, novobiocin, and cationic peptides.^[^ ^53^ ^]^ Hence, blockade on such TCSs has been severally proposed as strategies to curtail the antibiotics resistance.^[^ ^52^, ^54^ ^]^ However, our data showed that different antibiotics may controversially respond to Cpx signaling, whose activation can also sensitize bacteria to specific antibiotics. Despite the nitrofurantoin used in this study, other antibiotics that depend on activation by reducing the nitro groups, such as metronidazole, deserve further investigation as they are also highly possible to respond to *cpxA*/*R*‐controlled nitroreductase overexpression. In the present study, AMK was found to activate the Cpx signaling for enhancing nitroreductase expression and NIT prodrug activation. Considering the widespread of envelope stimuli in nature, for example other aminoglycoside antibiotics (Figure 6i), there are supposed to be a plethora of antibiotic or non‐antibiotic compounds (like ascorbic acid and etc.) with potential to manipulate nitroreductase expression and antibiotic susceptibility via Cpx signaling.^[^ ^55^ ^]^

In this study, we only focused on the synergistic interaction of AMK and NIT for eradication of uropathogens as a proof‐of‐principle because both drugs were routinely used in treatment for UTI. But after pharmacodynamical behavior of combination is well elucidated in the future, the combinations of NIT to AMK or other aminoglycosides are also of high potential to target other infections caused by any pathogen that possesses TCS *cpxA*/*R*.

## 4 Conclusion

In summary, the combination of AMK and NIT was found to synergistically eradicate Gram‐negative uropathogens in vitro and in vivo. Further mechanistic analysis revealed that Cpx signaling was activated in response to AMK‐mediated envelope stress, which constitutively stimulated the expression of bacterial nitroreductases via *soxS*/*marA* regulons. The overexpression of nitroreductases promoted the prodrug activation of NIT and accumulation of lethal ROS. This study highlighted the potential of AMK‐NIT combination in treatment for UTI and shed the light on the promising role of bacterial TCS in manipulating antibiotic susceptibility.

## 5 Experimental Section

### Bacterial Strains and Cultivation

The *E. coli* BW25113, *E. coli* CFT073, *K. pneumoniae* ATCC700603, *P. mirabilis* ATCC35659, *E. facalis* ATCC29212 were either from the lab collection or obtained from the third affiliated hospital of Sun Yat‐sen University. The corresponding isogenic mutants were constructed in the current study. All the bacterial strains used in this study are listed in the Table S1 (Supporting Information). Routine propagation of bacteria was in Luria‐Bertani (LB) medium and the antimicrobial susceptibilities of all tested strains were determined by Mueller‐Hinton (MH) broth, unless otherwise noted.

### Genetic Manipulation of Bacterial Strains

Multiple plasmids were constructed for genetic manipulation of bacterial strains in this study (Table S2, Supporting Information). The primers used for plasmid construction can be found in Table S3 (Supporting Information). The *nfsA*, *nfsB, marA, soxS, cpxA, cpxR* and *lon* genes in BW25113 and CFT073 were knocked out by *λ*Red recombination system,^[^ ^56^ ^]^ to obtain corresponding mutant strains. The low copy number plasmid, pGEN, was used to for complementation of genes. The *luxCDABE* genes were fused with promoter of *nfsA* and cloned into pUC plasmid to construct a reporter plasmid (pUC‐luxCDABE‐*nfsA*) for monitoring its expression. The plasmid pBAD24::CpxR‐HA carried *cpxR* gene fused with a HA tag was constructed for the Phos‐tag assay.

### Antimicrobial Susceptibility Testing and Checkerboard Assay

The minimum inhibitory concentrations (MIC) assay was performed to determine the antimicrobial susceptibility according to the standard protocol. The results were interpreted on basis of guidelines of the Clinical and Laboratory Standards Institute (CLSI). Briefly, both drugs were twofold diluted in medium and mixed with an equal volume of bacterial suspension (10^6^ CFU/mL) in microtiter plate. After incubation at 37 °C for 18 h, the MIC values were defined as the lowest concentrations of antibiotics with no visible growth of bacteria. To better understand the synergy between AMK and NIT, the checkerboard assay was conducted to determine the fractional inhibitory concentration index (FICI) as described previously with minor modifications.^[^ ^21^ ^]^ In brief, 100 µL of MH broth was first dispensed to each well of a microtiter plate with serial concentrations of AMK and NIT. Subsequently 5 × 10^5^ CFU mL^−1^ of bacteria was inoculated in the same plate and incubated at 37 °C for 18 h. After the incubation, the OD_600nm_ was determined using a microtiter plate reader (PerkinElmer, USA). The FICI was calculated by the formula as follows: FICI = (MIC_A_ combination/MIC_A_ alone) + (MIC_B_ combination/MIC_B_ alone). FICI of ≤0.5, 0.5–2, and >2 is defined as synergism, additive/ indifference and antagonism, respectively.^[^ ^57^ ^]^

### Time‐dependent Killing Assay

The bacterial culture to exponential phase were diluted in MH broth of 4 mL to an optimal concentration at 10^6^ CFU mL^−1^, which were subsequently treated with AMK, NIT or their combination for incubation at 1/2 MIC. At the time points 0, 3, 6, 9, and 24 h, 100 µL aliquot (*n* = 3) of each treatment was removed and plated for determination of bacterial survivors.

### Ethic Approval

This study was carried out in accordance with the recommendations of ethical guidelines of South China Agricultural University. All animal experimental protocols were reviewed and approved by the South China Agricultural University Institutional Animal Ethics Committee (2021C114).

### 5.1 mellonella Infection Model

The synergistic interaction between AMK and NIT was first elucidated in *G. mellonella* infection model. The larvae of *G. mellonella* were randomly divided into four groups (*n* = 10) and infected with UEPC CFT073 (10^5^ CFU, 10 µL) at the right posterior gastropoda. After 3 h post‐infection, larvae were treated with i) PBS (control), ii) AMK (7.5 mg kg^−1^), iii) NIT (3.75 mg kg^−1^), and iv) combination of AMK (3.75 mg kg^−1^) with NIT (1.875 mg kg^−1^) at the left posterior gastropoda. The survival of larvae in each group was monitored throughout the experiment for 3 days.

### Mouse Model of Acute UTI

A total of 24 female C57BL/6J mice of 6 weeks were randomly assigned into four groups (*n* = 6 per group) in ventilated cages and had ad libitum access to feed and water. At the day of infection, the mice were anesthetized with 1.5% pentobarbital sodium before being infected with UEPC CFT073 or corresponding mutants as indicated (10^8^ CFU, 50 µL). The infected animals were divided into four groups and respectively received i) PBS (control, 100 µL), ii) AMK (7.5 mg kg^−1^, 100 µL), iii) NIT (3.75 mg kg^−1^, 100 µL), and iv) combination of AMK (3.75 mg kg^−1^, 50 µL) with NIT (1.875 mg kg^−1^, 50 µL) at 12 h post infection for once. At the 3 days post the infection, the mice were sacrificed after anesthesia and the bladders of tested animals were removed. The bladders were homogenized in cold PBS using a bead beater (MP Biomedicals, USA) and the homogenized mixtures were then subjected for bacteria enumeration by plating.

### Biocompatibility Evaluation

Female C57BL/6J mice of 6 weeks randomly assigned to seven cages (7 per cage) in ventilated cages. Mice were acclimated for 3 d prior to biocompatibility test. At the day of challenge, the mice received either i) PBS (control, 100 µL) or ii) combination of AMK (3.75 mg kg^−1^, 50 µL) with NIT (1.875 mg kg^−1^, 50 µL). The body weights and survivals of mice in each group were consecutively recorded for 7 days. After day 9, the mice were sacrificed to collect kidney and liver for pathological study with H&E staining. In addition, serum samples (200‐300 µL per mouse) were also harvested from tested mice to exam the blood biochemical indices, including gamma‐glutamyltransferase (GGT), total bilirubin (T‐BILL), urea, aspartate transferase (AST), alanine transaminase (ALT), alkaline phosphatase (ALP), *α*‐amylase (*α*‐AMY), creatinine (CREA) with an automatic biochemical analyzer to verify whether the combinatorial therapy induces hepatic and renal damage.

### RNA‐seq and Transcriptomic Analysis

The *E. coli* BW25113 was grown in MH broth to the exponential phase, then subjected to co‐incubation with PBS, AMK, NIT, or AMK‐NIT combination (*n* = 3 per group) at sublethal concentration for 4 h. After the incubation, the bacterial cells were washed trice and the total RNA of each sample was were extracted using the OMEGA Total RNA Kit I (Omega, China). The library was constructed using an Illumina TruSeq RNA sample Prep Kit v2 (Illumina, USA) in accordance with previous protocol.^[^ ^58^ ^]^ In brief, mRNA was fragmented into lengths of 200–300 bp, thereafter the first and second strand cDNA were synthesized. The short cDNA fragments were purified and end repaired and tailed with single A (adenine) addition. Adapters were ligated to the A‐tailed cDNA fragments. In sequencing data analysis, short sequences (reads) were read by FANse2 software. By mapping to the reference sequence (https://www.ncbi.nlm.nih.gov/nuccore/NC_000913.3), the number of reads through the genetic regions estimate the level of gene expression. The differentially expressed genes were identified by Q‐value ≤0.01 and fold‐change ≥2. The GO enrichment analysis of differentially expressed genes was performed by ClueGO of Cytoscape application.

### Intracellular ROS Determination

ROS were measured by flow cytometry using 2′,7′dichlorodihydrofluorescein diacetate (DCFH‐DA). Exponential‐phase bacterial cultures were washed trice with cold PBS and re‐suspended in medium supplemented with NIT, AMK or their combination (*n* = 3 per group) at sublethal concentration for 1 h. Subsequently, the cells were washed twice with PBS and incubated with 10^−6^ M DCFH‐DA for 20 min at 37 °C. After that, the cells were washed twice with PBS, re‐suspended in 200 µL PBS, and the ungated events for each sample was determined by a BD Accuri C6 Plus flow cytometer (BD, USA).^[^ ^59^ ^]^

### Directed Evolution Assay

For the directed evolution assay, the *E. coli* BW25113 was primed by non‐lethal dose of AMK or NIT in a serial passage (*n* = 3 per group). The sub‐population in each serial passage was renamed as EA (evolution under AMK) or EN (evolution under NIT), then subjected to MIC and FICI test to observe whether bacteria altered their responses to the combination of AMK and NIT. The whole‐genome sequences of evolved mutants were determined by long‐read sequencing using Oxford Nanopore MinION (Oxford Nanopore Technologies, UK). SPAdes v3.8.7 was used for de novo assembly of sequencing data to profile the mutations.^[^ ^60^ ^]^

### RNA Isolation and RT‐qPCR

The total RNAs were extracted using OMEGA Total RNA Kit I (Omega, China). Reverse transcription was performed on 1 µg total RNA using GoldenstarRT6cDNA Synthesis Mix (TsingKe Biotech. China). The qRT‐PCR was performed with SYBR Master Mix (Vazyme, China). The primers used for RT‐qPCR were listed in Table S3 (Supporting Information). Relative expression values were obtained by the 2^–ΔΔCt^ method as elucidated previously.^[^ ^61^ ^]^

### Immunoblotting and Phos‐tag Assay

To determine the expression of NfsA, NfsB, SoxS and MarA at protein level, the western blotting was performed on either BW25113 or its Lon‐deficient mutant. After being challenged by AMK, NIT or their combination (*n* = 3 per group) for 1 h, cell pellets were washed with ice‐cold 10 mm Tris‐Cl (pH 6.8) buffer to prepare the cell lysates via sonication. A total of 200 µL lysate of each was combined with 100 µL of 3 × SDS loading buffer then heated by 95 °C for 5 min. After heating, 10 µL of aliquot was loaded onto a SDS‐PAGE gel. The proteins of NfsA and NfsB were separated by electrophoresis at 4 °C then transferred to a PVDF for immunoblotting.

The phosphorylation of bacterial two‐component system protein (CpxR and CpxR‐P) was determined by Phos‐tag assay as dated previously.^[^ ^62^ ^]^ Briefly, BW25113/pBAD24::CpxR‐HA were grown in MH broth in presence of AMK, NIT or their combination (*n* = 3 per group). The proteins of cell lysate were extracted as describe above and separated by Phos‐tag gel. The protein of target was transferred to a PVDF for immunoblotting. The blots were probed with indicated antibodies and the phosphorylation of proteins was quantified with ImageJ.

### Determination of Cellular NADH and NADPH

To estimate the cellular nitroreduction level, the consumption of cofactors, NADH and NADPH, were quantified. In brief, the bacterial cells were challenged by AMK, NIT or their combination (*n* = 3 per group) for 30 min, thereafter the cell pellets were collected after washing with PBS trice. Then the pellets were re‐suspended in precooled extraction buffer, where the cell lysates were harvested. The lysates were centrifuged at 10 000 *g* for 10 min at 4 °C to collect the supernatants. Finally, the cellular NADH and NADPH in the supernatants were estimated using commercial kits (Beyotime, China).

### Transcriptional Reporter Assay

The aforementioned pUC‐luxCDABE‐*nfsA* reporter plasmid was transformed into wild‐type or mutant *E. coli* to monitor the expression dynamic of NfsA in response to drug treatments. The bacteria were inoculated in LB with AMK‐NIT combination, where the luminescence and OD_600_ were both read after incubation of 6 h. The promoter activity was presented by luminescence (RLU) that normalized to OD_600_.

### In Vivo Imaging for Nitroreductase Expression

To investigate the modulation of bacterial nitroreductase by AMK, the expression of NfsA was monitoring using *E. coli* that carried aforementioned pUC‐luxCDABE‐*nfsA* reporter. Briefly, the bacteria were harvested in LB broth and infected mice bladders after being washed trice. Then, the tested mice received either i) PBS or ii) AMK‐NIT at dose mentioned in above section at 3 h post infection. Then the animals were anesthetized for probing nitroreductase expression using in vivo imaging system (IVIS, PerkinElmer, USA).

### Determination of Protein Aggregates

To demonstrate the application of AMK induced protein misfolding in bacteria, the protein aggregates were detected as a consequence of protein misfolding according to previous protocols.^[^ ^63^ ^]^ Namely, the bacterial cells were treated by AMK (1/2 MIC) or AMK in supplementation of chemical chaperone proline (0.5 mm) for 0.5 h, thereafter was washed with cold PBS for trice. The treated cells were stained using FITC (130 µm) for 15 min. After the incubation, the cells were washed and the protein aggregates were visualized using confocal microscopy (Leica, Germany).

### Electrophoretic Mobility Shift Assay

To verify the direct modulation of Cpx signaling on *soxS*/ *marA*, the electrophoretic mobility shift assay (EMSA) was performed as described previously. The *marbox* promoter was amplified and the CpxR protein was purified after expression in *E. coli*. Then, the purified DNA fragments of the promoter region was mixed with the serial dilutions of the CpxR proteins and kept in binding buffer (40 mm Tris‐HCl, 4 mm MgCl_2_, 100 mm NaCl, 10% glycerol, 2 mm dithiothreitol, 0.2 mg mL^−1^ bovine serum albumin and 1 mm EDTA) for 30 min at room temperature. Electrophoresis was carried out and stained with ethidium bromide to visualize the DNA bands.

### Statistical Analysis

Results were presented as means ± standard deviation (SD). The sample sizes (n) were described in the subsections for each assay. The statistical analysis was by performed using SPSS software (IBM, United States). Unless stated otherwise, statistical significance of comparison was assessed using the unpaired T‐test or one‐way ANOVA (* *p* < 0.05, ** *p* < 0.01, *** *p* < 0.001, ns: not significant).

## Conflict of Interest

The authors declare no conflict of interest.

## Author Contributions

H.R. and Z.Z. contributed equally to this work. H.R. and J.S conceptualized the study. Z.Z., Z.S., Y.W., Y.L., H.H., Z.Z., M.L., Q.H., and T.L performed the experiments. H.R., Z.Z., and X.L. analyzed the data. H.R. and Z.Z. visualized the data. H.R. and J.S. wrote and edited the manuscript. H.R., J.S., X.L., and Y.L. contributed to funding acquisition.

## Supporting information

Supporting Information

## Data Availability

The data that support the findings of this study are available on request from the corresponding author. The data are not publicly available due to privacy or ethical restrictions.
