# Supplementary material for: CpxA/R‐Controlled Nitroreductase Expression as Target for Combinatorial Therapy against Uropathogens by Promoting Reactive Oxygen Species Generation
Source: Adv Sci (Weinh). 2023 Jul 5;10(25):2300938. doi: 10.1002/advs.202300938 (PMC10477892; doi:10.1002/advs.202300938)
Supplement: Supplementary file 1 — Supporting Information [file ADVS-10-2300938-s001.pdf]

ADVANCED SCIENCE
Open Access

Supporting Information

for *Adv. Sci.*, DOI 10.1002/advs.202300938

CpxA/R-Controlled Nitroreductase Expression as Target for Combinatorial Therapy against Uropathogens by Promoting Reactive Oxygen Species Generation

*Hao Ren, Zixing Zhong, Shuang Zhou, Yiyang Wei, Yujiao Liang, Huiling He, Zijian Zheng, Mengyuan Li, Qian He, Tengfei Long, Xinlei Lian, Xiaoping Liao, Yahong Liu and Jian Sun**

Supporting Information

CpxA/R-controlled nitroreductase expression as target for combinatorial therapy against uropathogens by promoting reactive oxygen species generation

*Hao Ren†, Zixing Zhong†, Shuang Zhou, Yiyang Wei, Yujiao Liang, Huiling He, Zijian Zheng, Mengyuan Li, Qian He, Tengfei Long, Xinlei Lian, Xiaoping Liao, Yahong Liu, Jian Sun**

Dr. H. Ren, Z. Zhong, S. Zhou, Y. Wei, Y, Liang, H, He, Z. Zheng, M. Li, Q. He, T. Long, Dr. X. Lian, Prof. X. Liao, Prof. Y. Liu, Prof. J. Sun

Guangdong Laboratory for Lingnan Modern Agriculture, National Risk Assessment Laboratory for Antimicrobial Resistance of Animal Original Bacteria, College of Veterinary Medicine, South China Agricultural University, Guangzhou 510642, China

E-mail: jiansun@scau.edu.cn.

Dr. H. Ren, Z. Zhong, S. Zhou, Y. Wei, Y, Liang, H, He, Z. Zheng, M. Li, Q. He, T. Long, Dr. X. Lian, Prof. X. Liao, Prof. Y. Liu, Prof. J. Sun

Guangdong Provincial Key Laboratory of Veterinary Pharmaceutics, Development and Safety Evaluation, South China Agricultural University, Guangzhou 510642, China

Prof. X. Liao, Prof. Y. Liu, Prof. J. Sun

Jiangsu Co-Innovation Center for the Prevention and Control of Important Animal Infectious Disease and Zoonoses, Yangzhou University, Yangzhou 225009, China

†These authors contributed equally to this work.

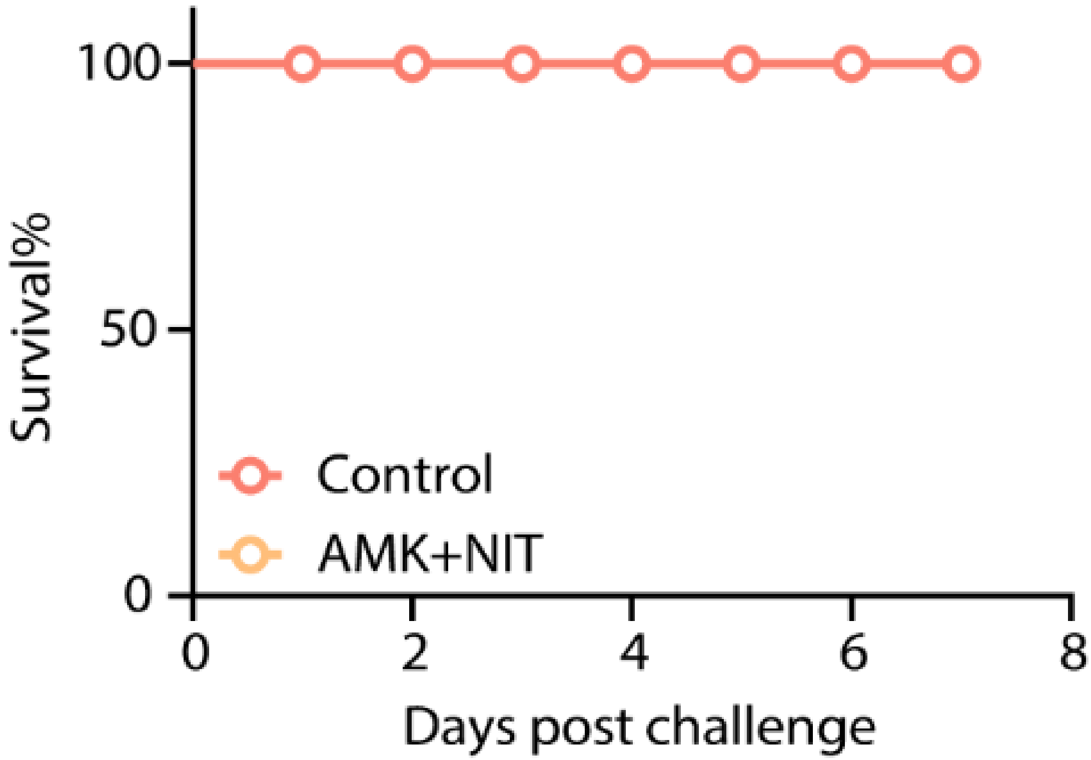

Figure S1 Survival curve of mice received PBS or AMK-NIT combination (n=7).

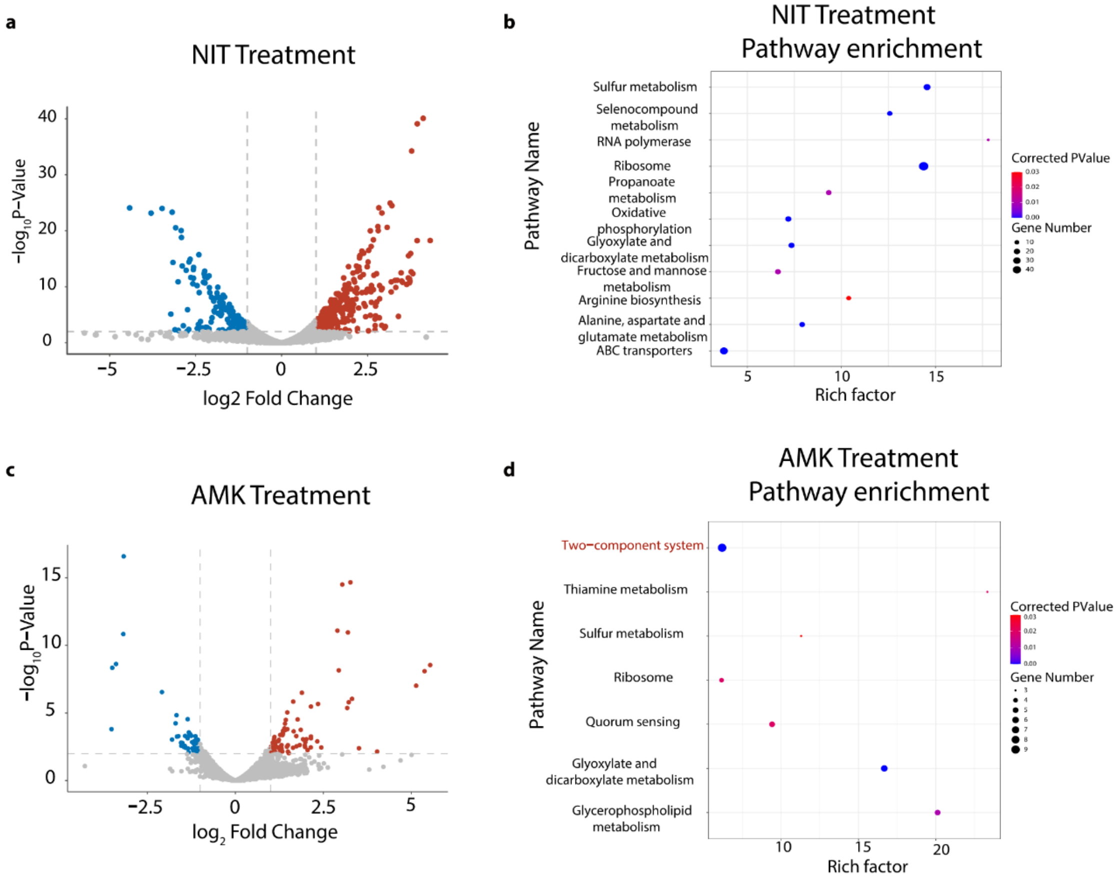

Figure S2 Transcriptomic analysis and pathway enrichments of bacteria treated by NIT (a, b) or AMK (c, d) alone.

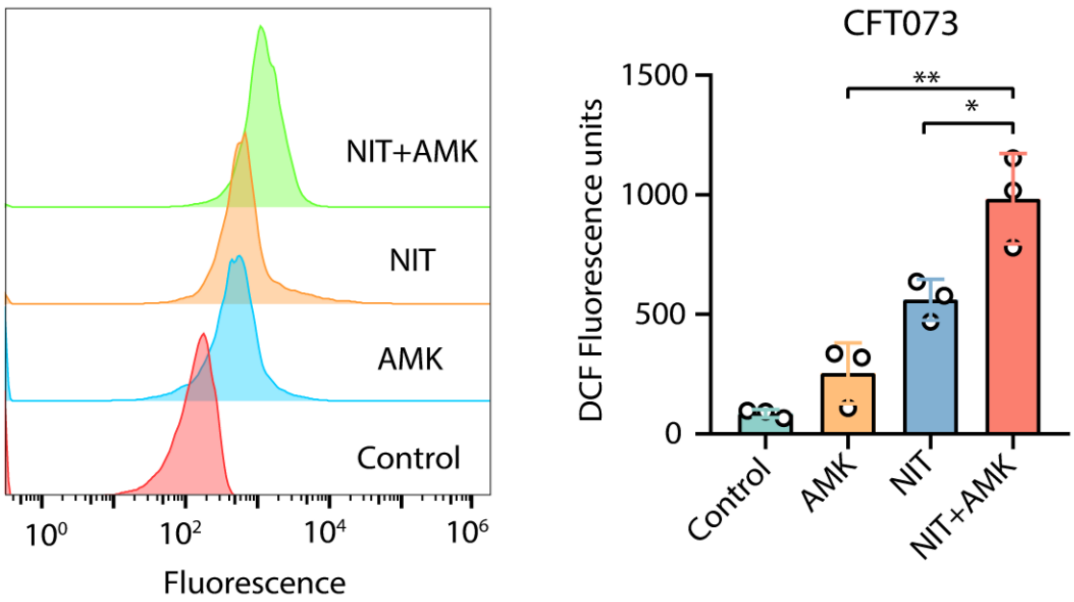

Figure S3 AMK-NIT combination promoted ROS generation in CFT073 (n=3).

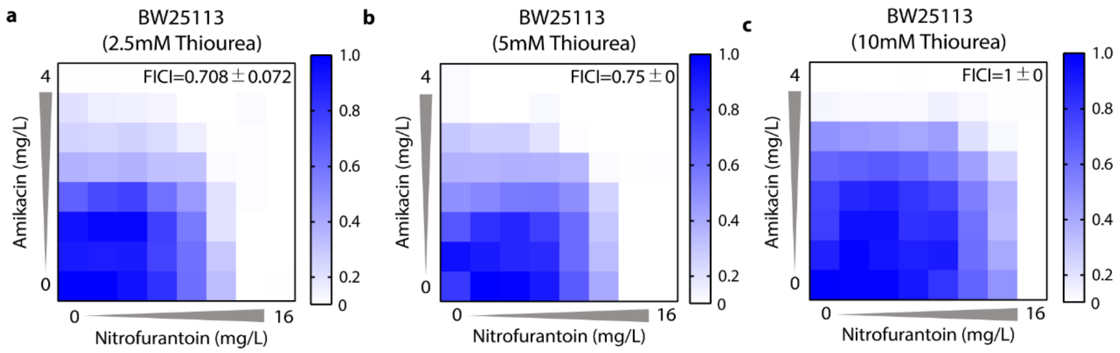

Figure S4 Checkerboard assays demonstrated the synergism between AMK and NIT was abolished (FICI of 0.5–2 was defined as additive or indifference effect) in presence of ROS-scavenging thiourea (a: 2.5mM; b: 5mM; c:10mM).

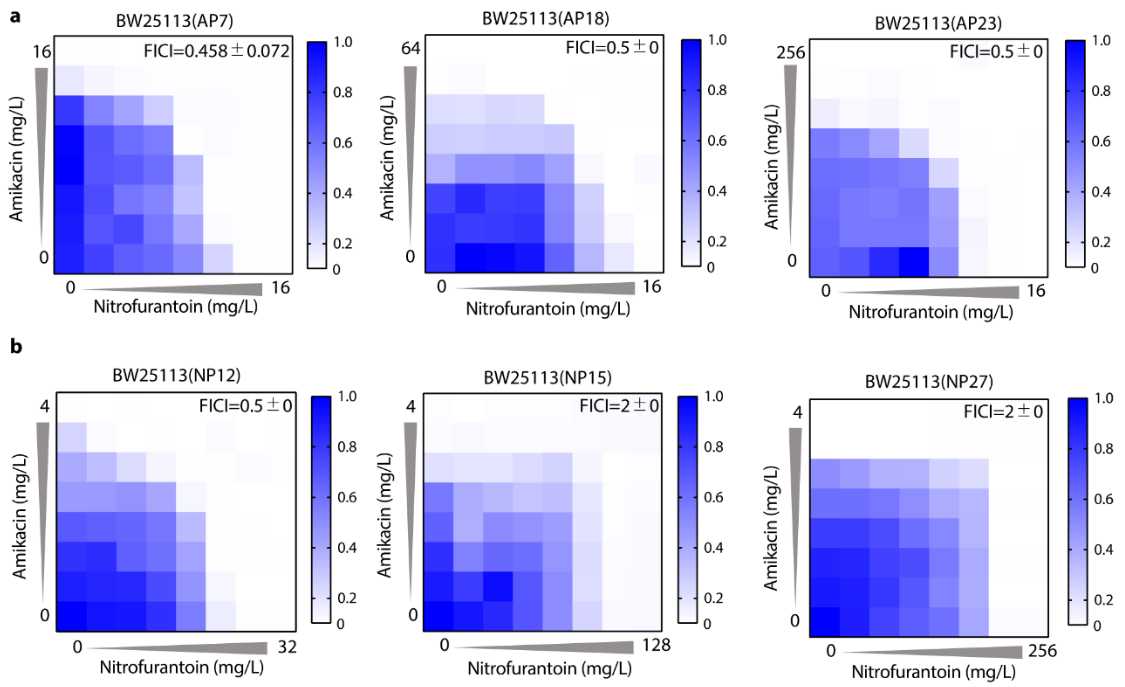

Figure S5 Acquisition of NIT (b) resistance but not the AMK (a) resistance during serial passage abolished their synergistic interaction (FICI of 0.5–2 was defined as additive or indifference effect).

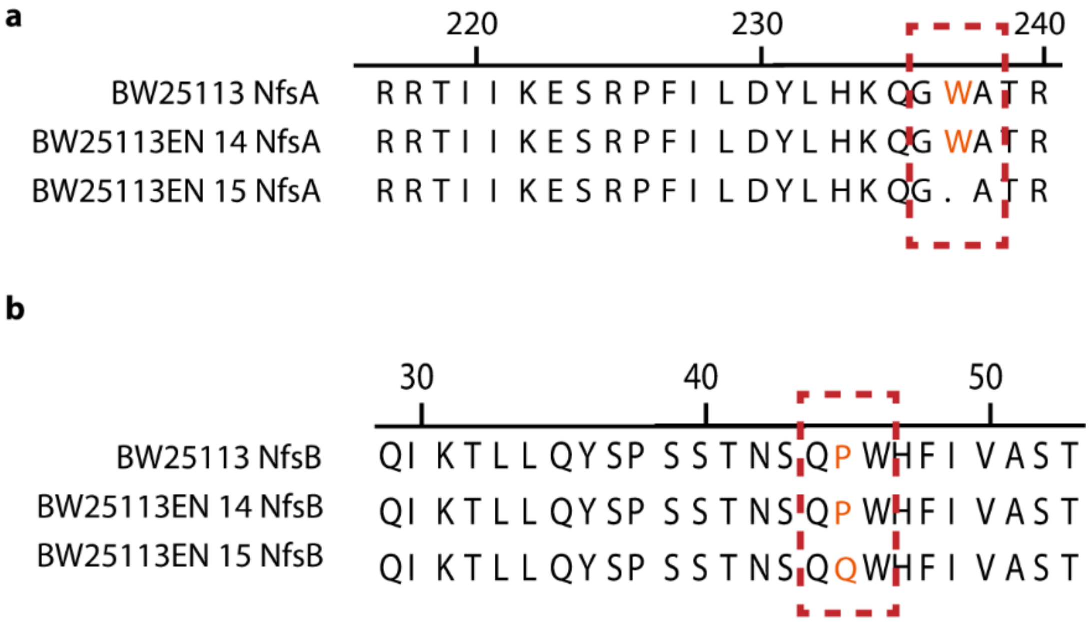

Figure S6 Mutations at bacterial major nitroreductases (a: *nfsA*; b: *nfsB*) were found to diminish the synergy between AMK and NIT.

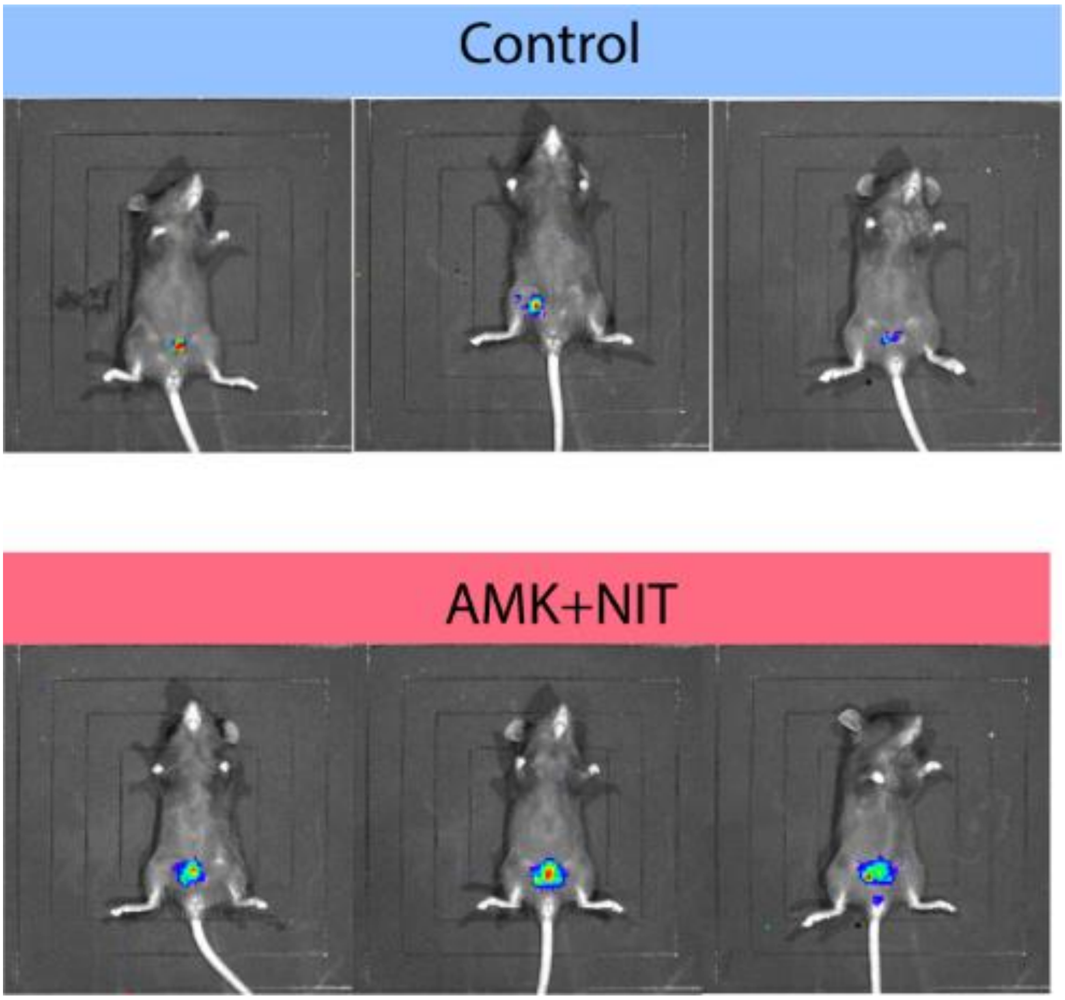

Figure S7 IVIS analysis (n=3) revealed that AMK-NIT combination stimulated the over-expression of nitroreductases *in vivo*.

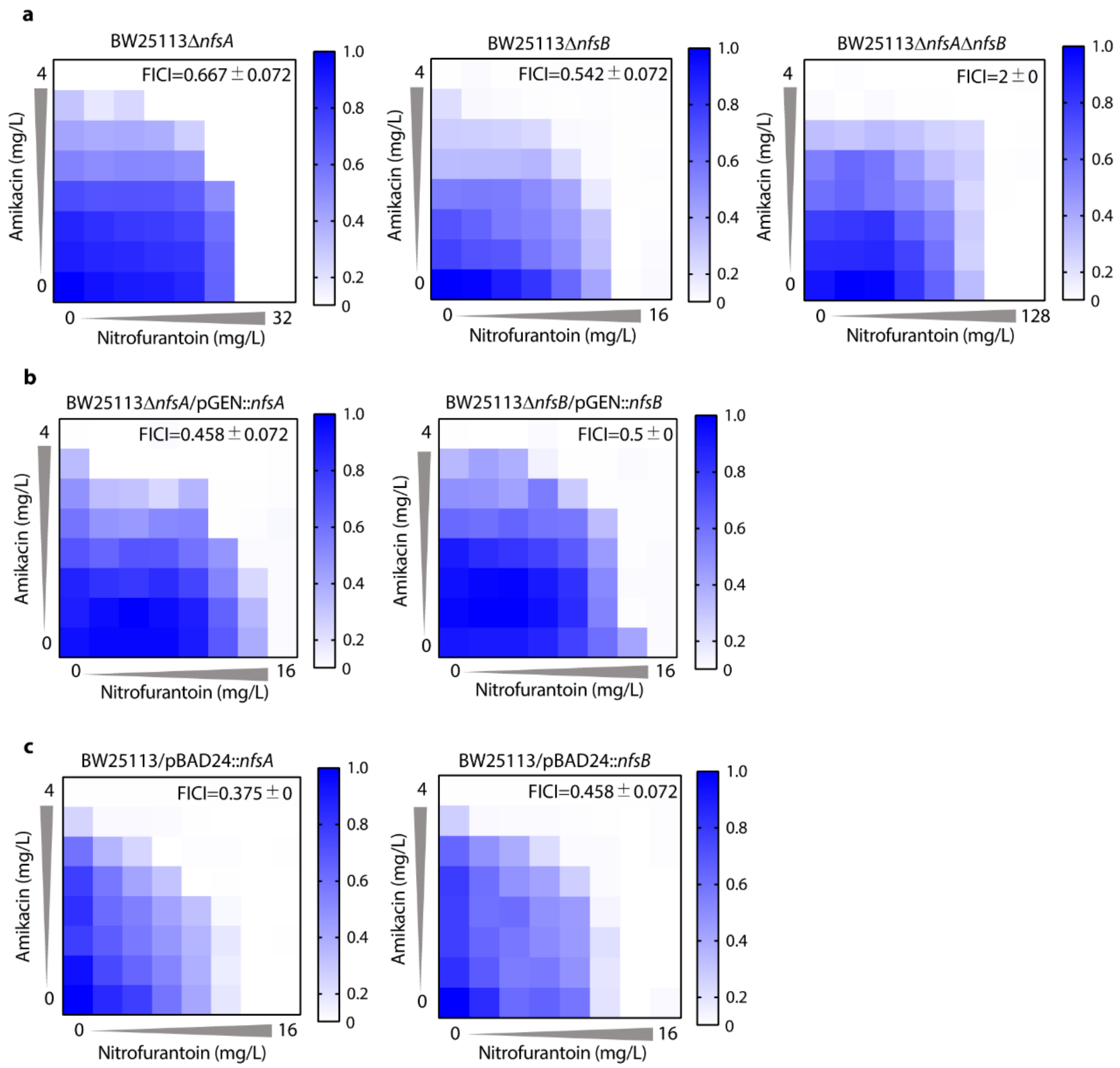

Figure S8 Checkerboard assays demonstrated that the deficiency in bacterial major nitroreductases abolished the synergism between AMK and NIT (a, b, c), which was compensated by plasmid-expressed *nfsA* or *nfsB* (b, c). FICI of 0.5–2 was defined as additive or indifference effect.

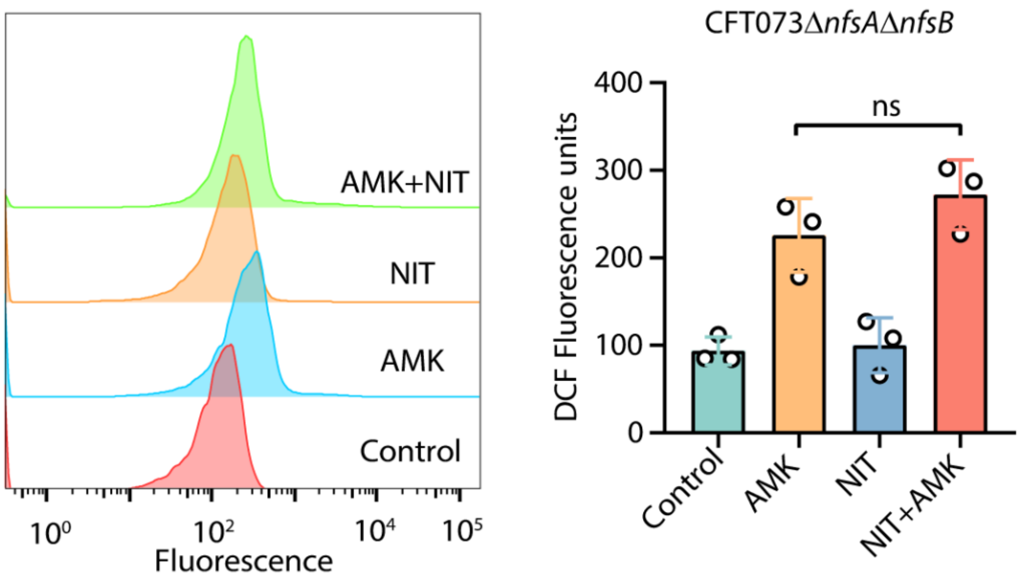

Figure S9 AMK-NIT combination failed to trigger the ROS overload in CFT073 without function of nitroreductases (n=3).

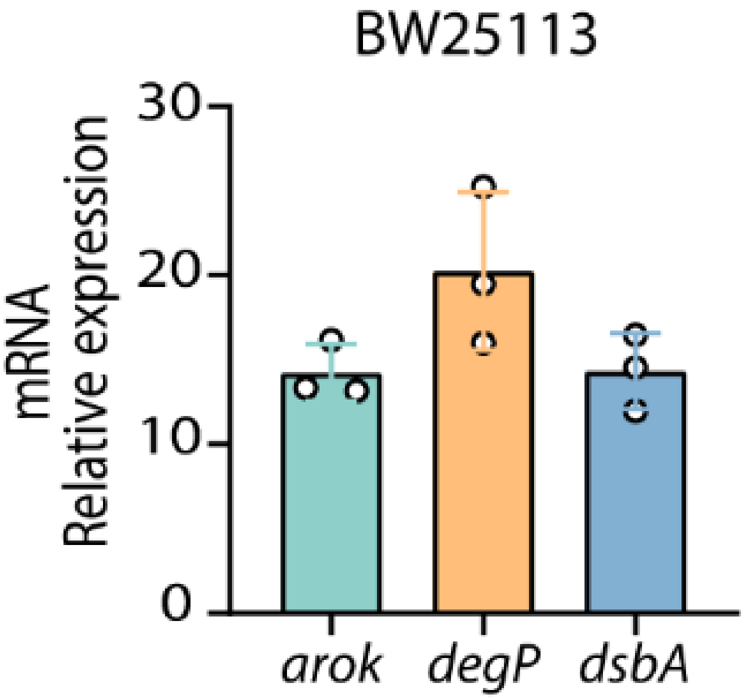

Figure S10 Genes (*aroK*, *degP* and *dsbA*) under control of *cpxA*/*R* for counteracting the envelope stress drastically increased in response to the combination of AMK and NIT (n=3).

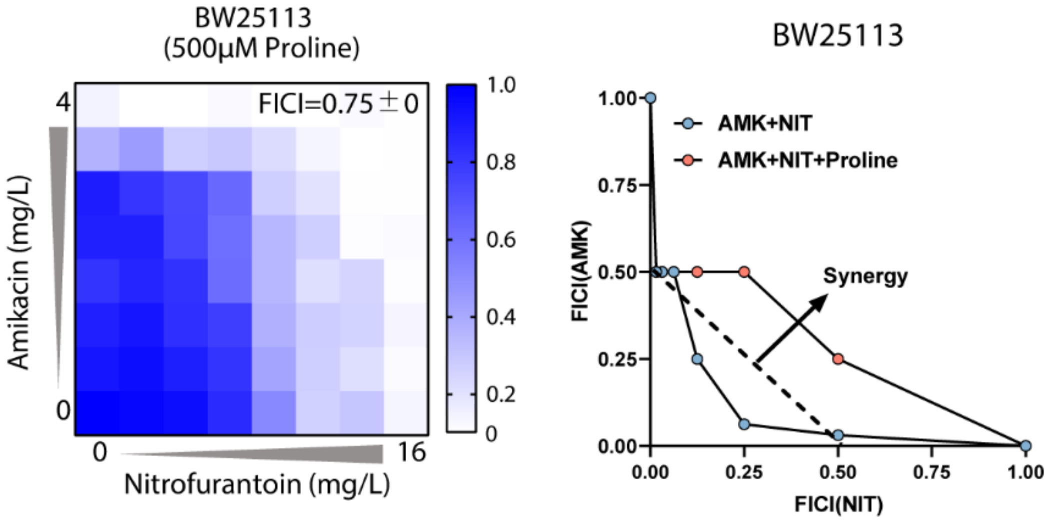

Figure S11 The chemical chaperone proline abolished the synergy between AMK and NIT (FICI of 0.5–2 was defined as additive or indifference effect).

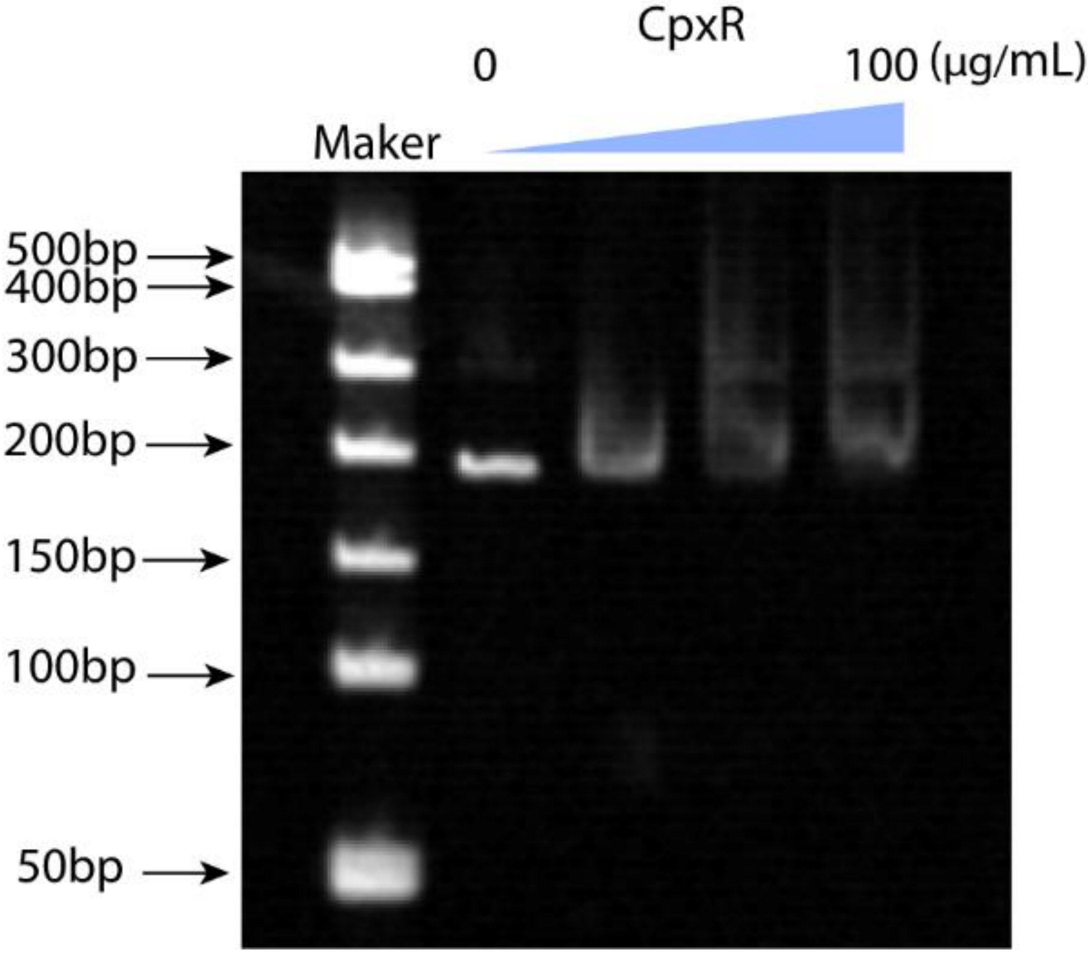

Figure S12 EMSA analysis demonstrated CpxR regulate *soxS*/*marA* expression by directly binding to *marbox*.

Table S1 Bacterial strains used in the current study.

Strains	Description	References
BW25113	Model strain	PMID:10829079
BW25113Δ*nfsA*	BW25113 with deletion of *nfsA*	This study
BW25113Δ*nfsB*	BW25113 with deletion of *nfsB*	This study
BW25113Δ*nfsA*Δ*nfsB*	BW25113 with deletion of *nfsA* and *nfsB*	This study
CFT073	Model UPEC strain from the third affiliated hospital of Sun Yat-sen University	This study
CFT073Δ*nfsA*Δ*nfsB*	CFT073 with deletion of *nfsA* and *nfsB*	This study
BW25113Δ*soxS*	BW25113 with deletion of *soxS*	This study
BW25113Δ*marA*	BW25113 with deletion of *marA*	This study
BW25113Δ*cpxA*	BW25113 with deletion of *cpxA*	This study
BW25113Δ*cpxR*	BW25113 with deletion of *cpxR*	This study
BW25113Δ*lon*	BW25113 with deletion of *lon*	This study
BW25113/pBAD24::*nfsA*	BW25113 carrying *nfsA* under the control of Para (AmpR)	This study
BW25113/pBAD24::*nfsB*	BW25113 carrying *nfsB* under the control of Para (AmpR)	This study
BW25113Δ*nfsA*/pGEN::*nfsA*	BW25113Δ*nfsA* with pGEN::*nfsA*	This study
BW25113Δ*nfsB*/pGEN::*nfsB*	BW25113Δ*nfsB* with pGEN::*nfsB*	This study
BW25113-P_{nfsA}-Lux	BW25113 carrying plasmid of P_{nfsA}-lux	This study

BW25113-P_{nfsA}-Lux/ pBAD24	BW25113-Lux carrying plasmid of pBAD24	This study
BW25113-P_{nfsA}-Lux/ pBAD24::*soxS*	BW25113-Lux carrying plasmid of pBAD24::*soxS*	This study
BW25113-P_{nfsA}-Lux/ pBAD24::*marA*	BW25113-Lux carrying plasmid of pBAD24::*marA*	This study
SoxS-P_{nfsA}-Lux	BW25113Δ*soxS* carrying plasmid of PnfsA-lux	This study
MarA-P_{nfsA}-Lux	BW25113Δ*marA* carrying plasmid of PnfsA-lux	This study
BW25113/pBAD24::*cpxR*-HA	BW25113 carrying plasmid of pBAD24::*cpxR-HA*	This study
BW25113Δ*nfsA/*pGEN::*nfsA-His*	BW25113Δ*nfsA* with pGEN::*nfsA-His*	This study
BW25113Δ*nfsB/*pGEN::*nfsB-His*	BW25113Δ*nfsB* with pGEN::*nfsB-His*	This study
BW25113Δ*lon/* pGEN::*soxS-His*	BW25113Δ *soxS* with pGEN::*soxS-His*	This study
BW25113Δ*lon/* pGEN::*marA-His*	BW25113Δ *marA* with pGEN::*marA-His*	This study

Table S2 Plasmids constructed in the current study.

Plasmids	Genotype or phenotype	References
pKD46	oriVpsc101(Ts); λRed recombinase expression vector	Datsenko KA, Wanner BL 2000,PMID:10829079
pCP20	Flp recombinase expression vector (CmRAmpR) (Ts)	Datsenko KA, Wanner BL 2000,PMID:10829079
pKD4	PCR template for λRed recombination system (KanR)	Datsenko KA, Wanner BL 2000,PMID:10829079
pBAD24	oriVp15A; Para (AmpR)NcoI site of expression vector pBAD24 changed to an NdeI site	This study
pBAD24::*nfsA*	pBAD24 carrying *nfsA* under the control of Para (AmpR)	This study
pGEN	oriP15A, low copy number plasmid (AmpR)	This study
pGEN::*nfsA*	pGEN carry *nfsA* with native promoter	This study
pGEN::*nfsB*	pGEN carry *nfsB* with native promoter	This study
pGEN::*nfsA-His*	pGEN carry *nfsA-His* with native promoter	This study
pGEN::*nfsB-His*	pGEN carry *nfsB-His* with native promoter	This study
pGEN::*soxS-His*	pGEN carry *soxS -His* with native promoter	This study
pGEN::*marA-His*	pGEN carry *marA -His* with native promoter	This study
P_{nfsA}-*lux*	The SoxS binding region of *ybjC-nfsA* was fused with luxCDABE	Paterson ES, Boucher SE & Lambert IB 2002, PMID:11741843

pBAD24::*soxS*	pBAD24 carrying *soxS* under the control of Para (AmpR)	This study
pBAD24::*marA*	pBAD24 carrying *marA* under the control of Para (AmpR)	This study

Table S3 Oligonucleotides primers used in the current study.

Primers	Sequence
Knock-nfsA-F	GAACCGTCCACCGCAATATTCACGTTCAGAAAGAGAAAAAGATATTGCAGCATTACACGTCTTGAG
Knock-nfsA-R	CAGACCTGGTCAAAAGCCCGCGTATCATACATCGACGTGGCGGTTGCTGACATGGGAATTAGCCA
Knock-nfsB-F	AGAATCTGAGAGGAAATAGCCGGGCAGATGCCCGGCAAGAGAGAAGCAGCATTACACGTCTTGAG
Knock-nfsB-R	CTTGTAATCTGCTGGCACGCAAAATTACTTTCACATGGAGTCTTTGCTGACATGGGAATTAGCCA
Knock-073nfsA-F	TGAACCATCCACCGCAATATTCACGTTCAGAAAGAGAAAAAGATATGCAGCATTACACGTCTTGAG
Knock-073nfsA-R	CAGACCTGGTCAAAAGCCCGCGTATCATACATCGACGTGGCAGTTGCTGACATGGGAATTAGCCA
Knock-073nfsB-F	CTTGTAATCTGCTGGCACGCAAAATTACTTTCACATGGAGTCTTTTGCAGCATTACACGTCTTGAG
Knock-073nfsB-R	AGAATCTGAGGGGAAATAGCCGGGCAGATGCCCGGCAAGGGAGAAGCTGACATGGGAATTAGCCA
Knock-soxS-F	GATGGAGCAATTACCCGCGCGGGAGTTAACGCGCGGGCAATAAAAGCAGCATTACACGTCTTGAG
Knock-soxS-R	CCAACAGATGAATTAACGAACTGAACACTGAAAAGAGGCAGATTTGCTGACATGGGAATTAGCCA
Knock-marA-F	ATTTGCTTAAGAAAGTCCTGCCGTAAACAAAAAAGAGGTATGACGGCAGCATTACACGTCTTGAG
Knock-marA-R	GAAAGTGGTTTCATGATTGCCTCAGTGACGTTGTCACGTTTTCAAGCTGACATGGGAATTAGCCA
Knock-cpxA-F	TAAAACCTTGCGTGGTCGCGGCTATCTGATGGTTTCTGCTTCATGGCAGCATTACACGTCTTGAG
Knock-cpxA-R	GCCGGATGCGGCGTAAACGCCTTATCCTGCCTGCAAATGCGAAGTGCTGACATGGGAATTAGCCA
Knock-cpxR-F	AGCGACGTCTGATGACGTAATTTCTGCCTCGGAGGTATTTAAACATGCAGCATTACACGTCTTGAG
Knock-cpxR-R	CAGCCAGAAGATGGCGAAGATGCGCGCGGTTAAGCTGCCTATCATGCTGACATGGGAATTAGCCA
Knock-Lon-F	AATTAGTTAACCAAAAAGGGGGGATTTTATCTCCCCTTTAATTTTTCCTCATTGCAGCATTACACGTCTTGAG
Knock-Lon-R	AAGGCTGGCAAGCCCGAATTAGCCTGCCAGCCCTGTTTTTATTAGTGCATTGCTGACATGGGAATTAGCCA
pKD46-F	CCCGTGACAGGTCATTCAGA

pKD46-R	GGCACTTTTCGGGGAAATGT
pCP20-F	GTCAGGTGGCACTTTTCGG
pCP20-R	GGTCTGACAGTTACCAATGC
Test-nfsA-F	ATGACGCCAACCATTGAACT
Test-nfsA-R	TTAGCGCGTCGCCCAACCCT
Test-nfsB-F	TTACACTTCGGTTAAGGTGA
Test-nfsB-R	ATGGATATCATTTCTGTCGC
Test-073nfsA-F	GAATTTCAACCAGGTGACCG
Test-073nfsA-R	TACCTGAGCCATTTTCTCGG
Test-073nfsB-F	CGTGGAATCTGGTGGTTGA
Test-073nfsB-R	CCATCGAGGTGGTGTGATC
RT-nfsA-F	TGCTCGGTGTCGTTGATACG
RT-nfsA-R	TCCAGCGGTTGATAGCTGTT
RT-nfsB-F	CCAGCACGGAAGAAGGTAAA
RT-nfsB-R	TTTGCACAGAACACCACGAC
RT-marA-F	CTGGAATCGCCACTGTCACT
RT-marA-R	CCTTCAGCTTTTGCGCGATT
RT-soxS-F	GATAATCGCTGGGAGTGCGA
RT-soxS-R	ACTTGCAACGAATGTTCCGC
RT-cpxA-F	TAAGTGGGCACCGCCAG
RT-cpxA-R	CGGGCGGTCAAACAGTA

RT-cpxR-F	CACACCAGACGCCTGTC
RT-cpxR-R	CGGTTAACTCCAGCGTTT
RT-16S-F	ATTAGATACCCTGGTAGTCCACGC
RT-16S-R	TTGCGGGACTTAACCCAAC
EcoRI-nfsA-F	CCGGAATTCTTAGCGCGTCGCCCAAC
SamI-nfsA-R	TCCCCCGGGATGACGCCAACCATTGAACT
EcoRI-nfsB-F	CCGGAATTCATGGATATCATTTCTGTCGC
SamI-nfsB-R	TCCCCCGGGTTACACTTCGGTTAAGGTGATGT
pBAD24-F	CACGGCAGAAAAGTCCACATTG
pBAD24-R	ACACTACCATCGGCGCTACG
Pro-nfsA-F	AAAGGGCAGATTGTGTCGACTCAGGCGTCCAGATTTTCTTT
Pro-nfsA-R	TGGATCGATAGCTGGTCGACTTAGCGCGTCGCCCAACCCTG
Pro-nfsB-F	AAAGGGCAGATTGTGTCGACATGAAACACCCTTTAGAAACCTTG
Pro-nfsB-R	TGGATCGATAGCTGGTCGACTTACACTTCGGTTAAGGTGATGTT
PnfsA-F	TTGTGATGGCTTCCATGTCGGCAGAGCCCTCAACGCGAGGGCTTG
PnfsA-R	TAATGAATGAAATTTTTTTAGTCATTATCTTTTTCTCTTTCTGAACGTG
Lux-F	ATGACTAAAAAAATTTCATTCATTATTAACGGC
Lux-R	TCCCCGAAAAGTGCCACCTGACGTCCTATCAAGTACCCAAATGTGG
soxS-F	CCGGAATTCATGTCCCATCAGAAAATTATTCAGG
soxS-R	GCGATGACGTCTTACAGGCGGTGGCGATAA
pBAD-soxS-F	TTGGGCTAGCAGGAGGAATTATGTCCCATCAGAAAATTATTCAGGAT

pBAD-soxS-R	CCCGGGTACCATGGTGAATTAATTACAGGCGGTGGCGATAA
pBAD-marA-F	TTGGGCTAGCAGGAGGAATTATGTCCAGACGCAATACTGAC
pBAD-marA-R	CCCGGGTACCATGGTGAATTCACGTTTTCAACTAGCTGTTG
cpxR-HA-F	TTGGGCTAGCAGGAGGAATTATGAATAAAATCCTGTTAGTTGATGATGAC
cpxR-HA-R	CCGGGTACCATGGTGAATTCTCAAGCGTAATCTGGAACATCGTATGGGTATGAAGCAGAAACCATCAGATAGC
NfsA-His-F	CACGCTTTTCGTTGGGATCTTCAGGCGTCCAGATTTTCTT
NfsA-His-R	AATTTTAGTGGTGATGGTGATGATGGCGCGTCGCCCAACCCTG
NfsB-His-F	CACGCTTTTCGTTGGGATCTATGGATAAGCAATCACTGCAC
NfsB-His-R	AATTTTAGTGGTGATGGTGATGATGCACTTCGGTTAAGGTGATGTT
SoxS-His-F	ACGCTTTTCGTTGGGATCTTTATCGAGCCGTATACCGTTC
SoxS-His-R	TTTAGTGGTGATGGTGATGATGCAGGCGGTGGCGATAATC
MarA-His-F	CACGCTTTTCGTTGGGATCTTGGTGGTTGTTATCCTGTGT
MarA-His-R	TTTTAGTGGTGATGGTGATGATGGCTGTTGTAATGATTTAATGGATGT
